# Single-cell multi-omics deciphers the myofibro-inflammatory program of cancer-associated fibroblasts in triple-negative breast cancer

**DOI:** 10.1038/s41420-026-03255-z

**Published:** 2026-07-11

**Authors:** Min Li, Jun Lin, Changwei Yang, Jiaqi Yang, Caiqin Mo, Zhishuo Zhang, Anping Liu, Wei Lin, Lin lin, Wanglong Li, Yiqiu Wang, Xinhuang Hou

**Affiliations:** 1 Department of Thyroid and Breast Surgery, The First Affiliated Hospital, Fujian Medical University, Fuzhou, China; 2 Department of Breast Surgery, Maternal and Child Health Care Hospital of Pingtan Comprehensive Pilot Zone, Pingtan, China; 3 Trauma Center and Emergency Surgery Department, The First Affiliated Hospital, Fujian Medical University, Fuzhou, China; 4 Department of Nutrition and Food Safety, School of Public Health, Fujian Medical University, Fuzhou, P.R. China; 5 Department of Thyroid and Breast Surgery, National Regional Medical Center, Binhai Campus of the First Affiliated Hospital, Fujian Medical University, Fuzhou, China; 6 Department of Orthopaedic Surgery, National Regional Medical Center, Binhai Campus of the First Affiliated Hospital. Fujian Medical University, Fuzhou, China; 7 Department of Vascular Surgery, The First Affiliated Hospital, Fujian Medical University, Fuzhou, China; 8 Department of Operating Room, the First Affiliated Hospital, Fujian Medical University, Fuzhou, China; 9 Department of Vascular Surgery, National Regional Medical Center, Binhai Campus of The First Affiliated Hospital, Fujian Medical University, Fuzhou, China; 10 Department of Urology, Urology Research Institute, The First Affiliated Hospital, Fujian Medical University, Fuzhou, China; 11 Department of Urology, National Regional Medical Center, Binhai Campus of the First Affiliated Hospital, Fujian Medical University, Fuzhou, China; 12 Department of Epidemiology and Health Statistics, School of Public Health, Fujian Medical University, Fuzhou, China

**Keywords:** Gene expression, Cancer genomics

## Abstract

Triple-negative breast cancer (TNBC) is an aggressive subtype associated with poor prognosis and limited therapeutic options, largely due to its unique tumor microenvironment (TME). Cancer-associated fibroblasts (CAFs) critically influence tumor progression and metastasis, yet their functional heterogeneity in TNBC remain poorly understood. An integrated multi-omic analysis was conducted using single-cell RNA sequencing and single-cell ATAC sequencing from TNBC and hormone receptor-positive/HER2-negative (HR + HER2-) breast cancers. The signaling axis was validated critically regulates both TNBC progression and CAF subtype switching. We identified a distinct CAF subpopulation, termed my_iCAFs, characterized by co-expression of myofibroblastic (*FAP*, *ACTA2*) and inflammatory markers (*CXCL12*), significantly enriched within the TNBC TME. My_iCAFs possess elevated activity of pathways involved in epithelial-mesenchymal transition, PI3K/AKT signaling, and pro-inflammatory TNF/NF-κB signaling. Multi-omic integration pinpointed FOSB as a central transcription factor whose expression and chromatin accessibility were selectively enhanced in my_iCAFs, potentially regulated by tumor-derived *CXCL8* signaling via syndecan receptors. Also, we found that the FOSB-HES1 axis can effectively activate the transition of CAFs into my_iCAFs. This study reveals a novel FOSB-driven myofibroinflammatory CAF subtype prominently enriched in TNBC, suggesting crucial roles in tumor aggressiveness and highlighting potential therapeutic targets within the stromal compartment of this challenging breast cancer subtype.

## Introduction

Breast cancer remains a significant global health challenge, characterized by substantial clinical and molecular heterogeneity that dictates therapeutic strategies and patient prognosis [1]. This heterogeneity extends beyond the tumor cells themselves to encompass the complex tumor microenvironment (TME), a dynamic ecosystem comprising various stromal cells, immune cells, vasculature, and extracellular matrix components [2, 3]. Growing evidence highlights the TME as an active driver—not just a passive bystander—in all stages of tumorigenesis, from initiation to metastasis and therapy resistance [4]. Understanding the intricate interplay between cancer cells and their surrounding microenvironment is therefore paramount for developing more effective diagnostic and therapeutic interventions against this pervasive disease [5].

Among the diverse subtypes of breast cancer, Triple-Negative Breast Cancer (TNBC) presents a particularly formidable clinical challenge [6]. Defined by the absence of estrogen receptor (ER), progesterone receptor (PR), and human epidermal growth factor receptor 2 (HER2) amplification, TNBC accounts for approximately 15–20% of cases and is associated with aggressive clinical behavior, higher rates of early relapse, and poorer overall survival compared to other subtypes, such as the more common Hormone Receptor-positive/HER2-negative breast cancers (HR + HER2- BC) [7, 8]. The lack of well-defined molecular targets renders TNBC ineligible for endocrine or HER2-targeted therapies, leaving cytotoxic chemotherapy as the primary systemic treatment option. It is increasingly recognized that the distinct biological characteristics of TNBC are profoundly influenced by its unique TME composition. Specifically, the stromal compartment, largely orchestrated by Cancer-Associated Fibroblasts (CAFs), exhibits notable differences between TNBC and other subtypes, yet a comprehensive understanding of how CAF heterogeneity contributes to the aggressive phenotype of TNBC remains incomplete [9, 10].

Single-cell technologies empower high-resolution dissection of CAF heterogeneity, providing deeper insights into the TME [11]. This study employs integrated single-cell RNA sequencing (scRNA-seq) and single-cell assay for transposase-accessible chromatin using sequencing (scATAC-seq) to comprehensively characterize CAFs in TNBC and HR + HER2- BC [12]. Our study pursued three key objectives: (1) to delineate the landscape of CAF heterogeneity between TNBC and HR + HER2- breast cancers, (2) to elucidate TNBC-specific CAF-tumor cell interactions and their underlying regulatory mechanisms, and (3) to experimentally validate these functional CAF subsets. Our integrated analysis reveals substantial heterogeneity in breast cancer CAFs and identifies a novel my_iCAF subpopulation displaying hybrid features - co-expressing myofibroblastic markers (*FAP*, *ACTA2*) and inflammatory factors (*CXCL12*) - that is preferentially enriched in TNBC TME. Mechanistically, we demonstrate that tumor-derived *CXCL8* promotes my_iCAF differentiation through activation of the transcription factor FOSB. These findings illuminate the complex stromal diversity driving TNBC progression and position my_iCAFs as both a defining characteristic of TNBC microenvironment and a potential therapeutic target for this aggressive disease [13].

## Results

### Comprehensive cellular landscape of breast tumor microenvironments

**Fig. 1:**
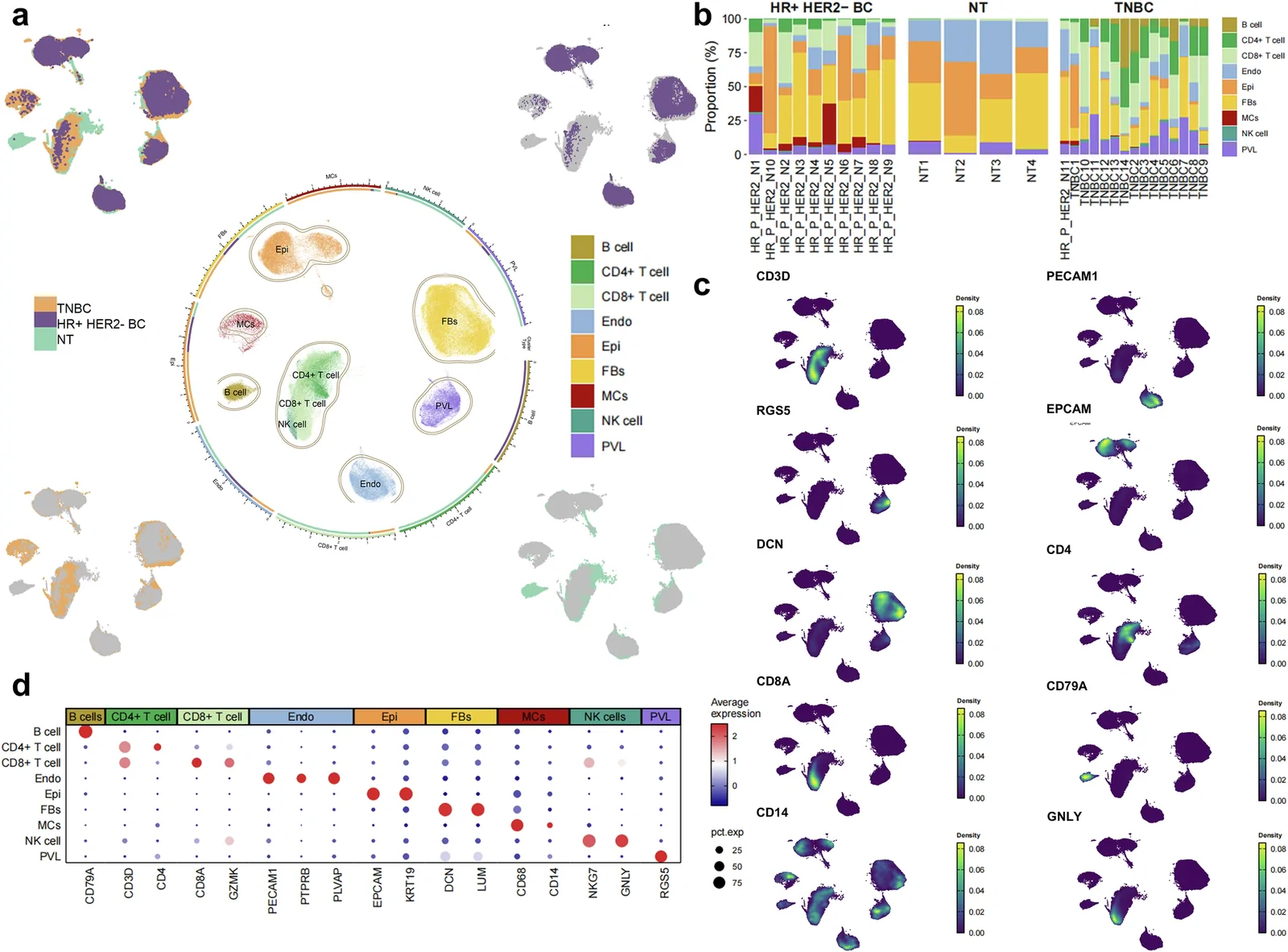
Cellular Composition of the Breast Tumor Microenvironment Revealed by Single-Cell RNA Sequencing. **a** UMAP embedding of 128,875 high-quality single cells isolated from TNBC, HR + HER2- BC, and NT. Cells are color-coded based on annotated major lineages. Key: Epi, Epithelial; Endo, Endothelial; PVL, Pericyte; FBs, Fibroblasts; MCs, Myeloid Cells. Lymphoid lineages (B cell, CD4 + T cell, CD8 + T cell, NK cell) are also indicated. **b** Stacked bar chart displaying the relative proportions of major cell lineages within each individual patient sample, categorized by tissue origin (TNBC, HR + HER2-, NT). **c** UMAP feature plots illustrating the normalized expression patterns of canonical marker genes used for cell type identification. **d** Dot plot summarizing the scaled average expression (color intensity) and the fraction of cells expressing (dot size) selected marker genes across all identified cell lineages, confirming cell type annotations. UMAP Uniform Manifold Approximation and Projection, TNBC Triple-Negative Breast Cancer, Hormone Receptor-positive/HER2-negative breast cancer, HR + HER2- BC; NT normal adjacent breast tissue.

Following stringent initial quality control and data filtering processes, a total of 128,875 individual cells were retained and subjected to comprehensive downstream sc-RNA seq analysis. Unsupervised clustering and subsequent cell type annotation, guided by canonical marker gene expression, successfully delineated eight principal cell populations within the analyzed breast tissue samples. These populations encompassed B lymphocytes, CD4 + T lymphocytes, CD8 + T lymphocytes, epithelial cells, endothelial cells, pericytes, various myeloid cell lineages, and natural killer cells. The overall cellular architecture and the relationships between these identified cell types were visualized using UMAP, which provided a global representation of the cellular heterogeneity across all samples. Furthermore, distinct UMAP projections were generated to specifically illustrate the cellular distributions within samples derived from TNBC, NT, and HR + HER2-BC subtypes, allowing for a visual comparison of the cellular composition associated with different breast cancer subtypes (Fig. 1a). To quantify the relative abundance of each major cell type across the different samples and conditions, compositional analyses were performed, and the resulting cell type proportions for each individual sample were graphically represented (Fig. 1b). The specificity of key marker genes used for cell type identification was further validated by visualizing their expression patterns overlaid onto the UMAP embedding, demonstrating distinct expression profiles largely confined to their respective annotated cell clusters (Fig. 1c). Complementing the UMAP visualization, a dot plot was constructed to quantitatively depict the expression levels and the percentage of cells expressing selected canonical marker genes within each identified cell population, providing robust confirmation of the assigned cell identities across the dataset (Fig. 1d). This initial characterization established the diverse cellular ecosystem present within the TME of TNBC and HR + HER2- BC, setting the stage for more detailed investigations into specific cell populations.

### Identification and molecular characterization of distinct CAF subpopulations

**Fig. 2:**
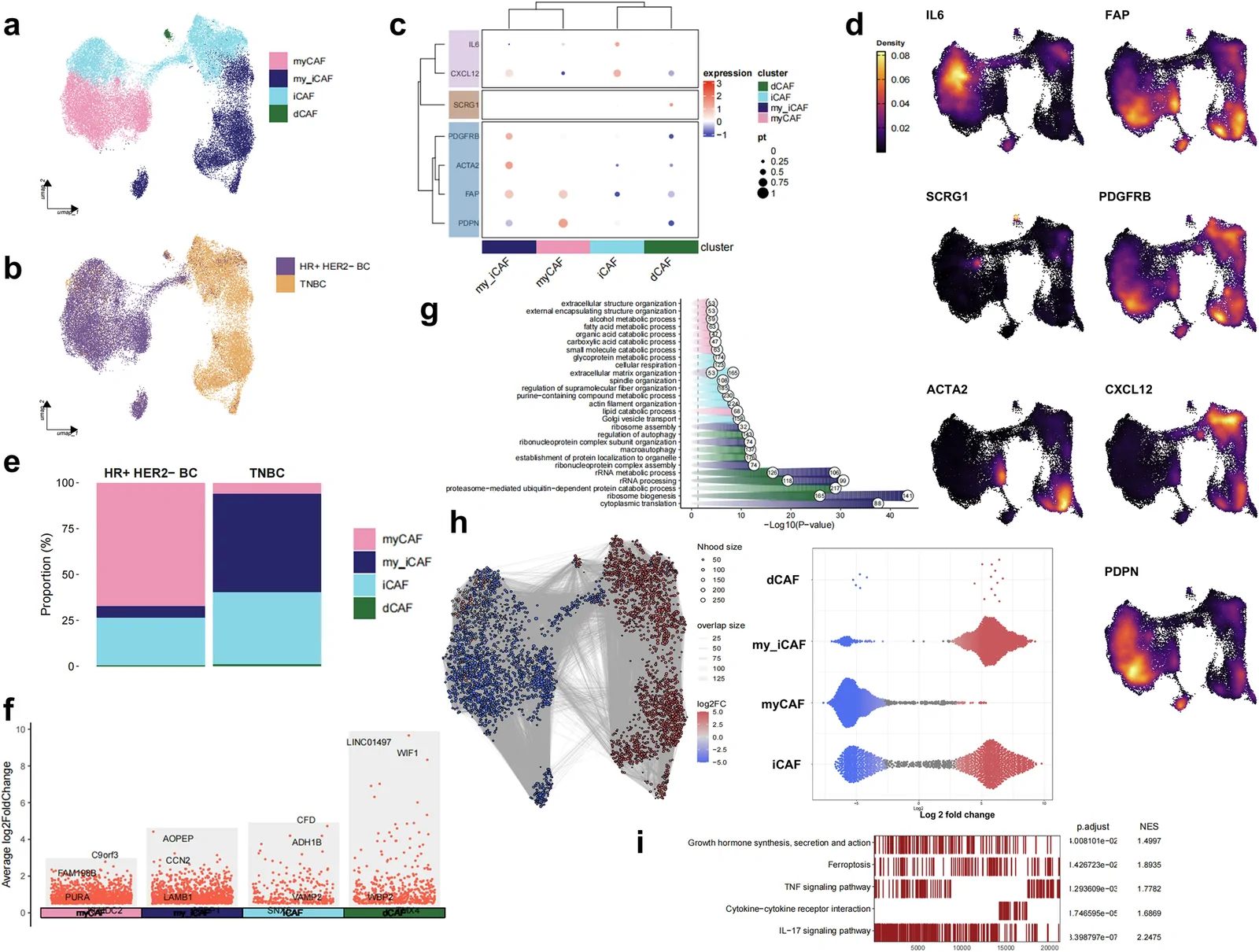
Heterogeneity and Subtyping of CAFs. **a** UMAP embedding of computationally isolated fibroblasts, revealing four distinct subpopulations: inflammatory CAFs (iCAFs), myofibroblastic CAFs (myCAFs), detached CAFs (dCAFs), and a mixed-phenotype my_iCAF population. **b** UMAP embeddings showing the distribution of CAF subpopulations segregated by tumor subtype of origin (TNBC vs. HR + HER2- BC). **c** Dot plot depicting scaled expression levels and percentage of expressing cells for defining marker genes across the four CAF subtypes. **d** UMAP feature plots showing the expression distribution of CAF subtype markers. **e** Bar chart illustrating the proportional representation of CAF subtype. **f** Volcano plots highlighting differentially expressed genes identified from pairwise comparisons between CAF subtypes. **g** Bar chart summarizing results from KEGG pathway enrichment analysis of genes significantly upregulated in each CAF subtype. Representative enriched pathways are listed (e.g., Extracellular structure organization for myCAFs; Cellular respiration for iCAFs; Cytoplasmic translation for my_iCAFs; Regulation of autophagy for dCAFs). **h** Differential abundance testing using Milo, visualizing neighborhood log-fold changes comparing TNBC and HR + HER2- BC conditions. **i** Gene Set Enrichment Analysis enrichment plots significantly enriched in the my_iCAF subtype compared to all other CAF subtypes (e.g., TNF signaling via NF-kB, Growth hormone synthesis, secretion and action). UMAP, Uniform Manifold Approximation and Projection; TNBC Triple-Negative Breast Cancer, Hormone Receptor-positive/HER2-negative breast cancer, HR + HER2- BC, CAF Cancer-Associated Fibroblasts.

To gain deeper insights into the heterogeneity within the stromal compartment, cells initially annotated as fibroblasts based on the global analysis were computationally isolated and subjected to a dedicated secondary analysis pipeline. This involved re-normalization of expression data, dimensionality reduction, and refined clustering specifically for the fibroblast population. This focused approach successfully resolved four distinct subpopulations of CAFs, herein designated as iCAFs, myCAFs, dCAFs, and a novel subtype exhibiting characteristics of both inflammatory and myofibroblastic lineages, termed my_iCAFs (Fig. 2a, b). The molecular identity of these CAF subtypes was defined by distinct gene expression signatures. Differential gene expression analysis revealed specific marker genes characterizing each subpopulation: iCAFs were distinguished by high expression of immunomodulatory factors such as *IL6* and *CXCL12*; dCAFs were uniquely characterized by *SCRG1* expression; myCAFs exhibited elevated levels of matrix-remodeling and activation markers including *FAP* and *PDPN*; whereas the my_iCAF subtype displayed a hybrid signature prominently featuring *CXCL12*, *ACTA2*, *FAP*, and *PDGFRB* (Fig. 2c). The specific distribution and expression intensity of these defining marker genes across the identified CAF subclusters were visualized on the fibroblast-specific UMAP plot (Fig. 2d). Notably, the my_iCAF subpopulation was markedly enriched in TNBC samples compared to HR + HER2- BC samples, while the proportion of myCAFs showed a corresponding significant reduction in TNBC (Fig. 2e). Further characterization involved identifying the top three most highly expressed genes within each CAF subtype, providing additional insights into their potential functional roles (Fig. 2f). To elucidate the biological pathways associated with these distinct CAF phenotypes, KEGG pathway enrichment analysis was performed on the upregulated genes specific to each subtype. This analysis revealed significant functional divergence: myCAFs showed enrichment in pathways related to “extracellular structure organization,” consistent with their myofibroblastic nature; iCAFs were enriched in pathways such as “cellular respiration,” suggesting distinct metabolic activity; my_iCAFs exhibited enrichment in “cytoplasmic translation,” possibly indicating high protein synthesis activity; and dCAFs showed enrichment in pathways including “regulation of autophagy” (Fig. 2g). Complementary differential abundance testing using Milo confirmed the significant enrichment of the my_iCAF population and the concurrent depletion of the myCAF population within the TNBC relative to the HR + HER2- BC, strongly suggesting that my_iCAFs represent a CAF phenotype particularly associated with TNBC, serving as the key CAF of TNBC (Fig. 2h). To further probe the functional characteristics of the TNBC-enriched my_iCAF subtype, Gene Set Enrichment Analysis was conducted comparing my_iCAFs against the other identified CAF subtypes. This analysis demonstrated significant enrichment of hallmark pathways such as “TNF signaling via NF-kB” and “Growth hormone synthesis, secretion, and action” within the my_iCAF population, underscoring their potential dual capacity to promote both pro-inflammatory responses and tumor growth-related processes within the TNBC microenvironment (Fig. 2i).

### Functional pathway activity and TF regulation within CAF subtypes

**Fig. 3:**
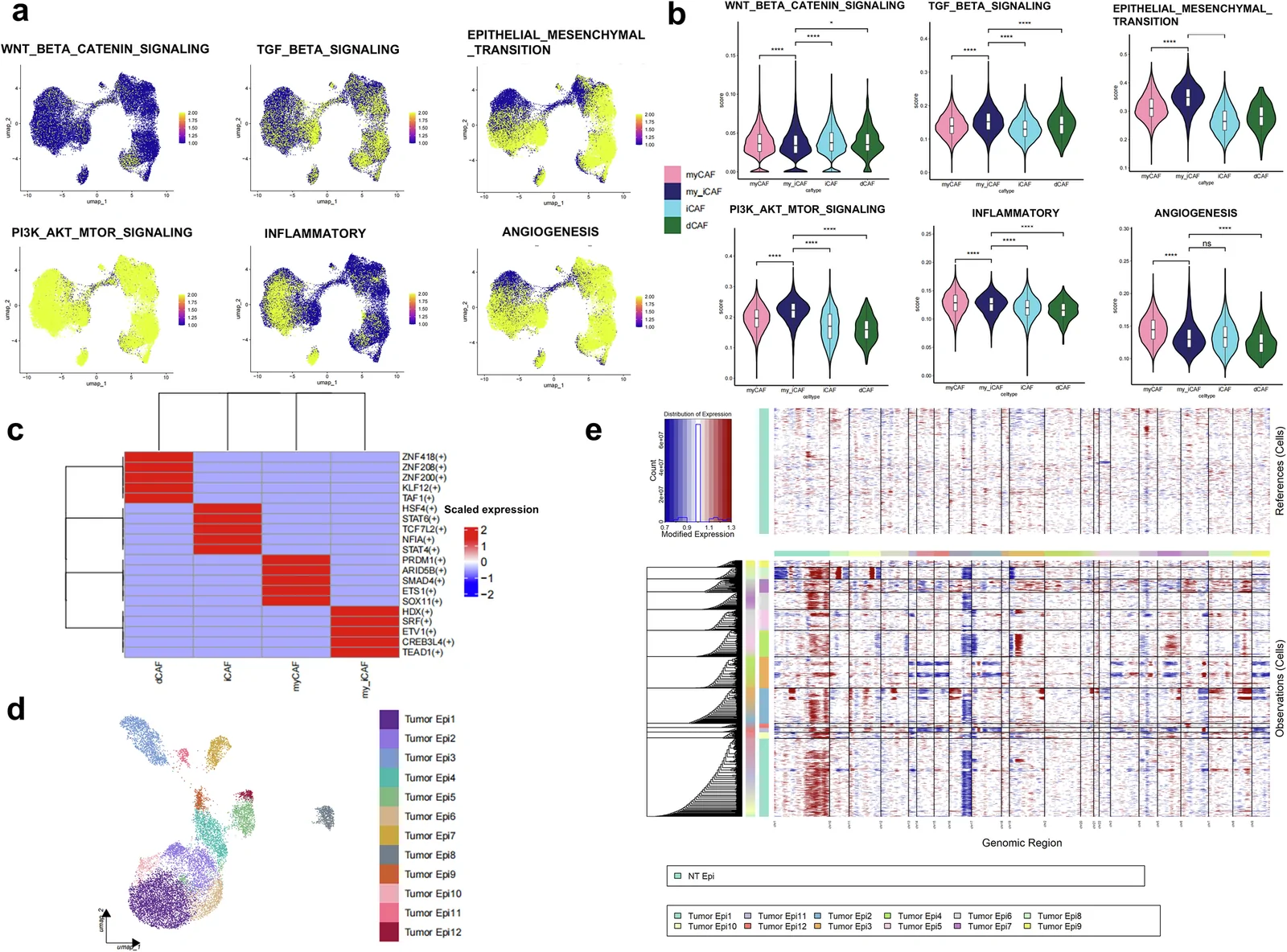
Functional States, Regulatory Networks of CAFs, and Tumor Cell Identification. **a** UMAP embeddings of CAF subpopulations overlaid with UCell enrichment scores, visualizing the activity distribution of selected Hallmark pathways (TGF_BETA_SIGNALING, EPITHELIAL_MESENCHYMAL_TRANSITION, PI3K_AKT_MTOR_SIGNALING, etc.). **b** Violin plots comparing UCell enrichment scores for the indicated Hallmark pathways across the four CAF subtypes. Statistical significance between groups is denoted. **c** Heatmap illustrating the activity scores of top variable transcription factor regulons inferred by pySCENIC across the CAF subtypes. **d** UMAP embedding of isolated epithelial cells following subclustering, revealing 12 distinct epithelial subpopulations derived from TNBC and HR + HER2- BC samples. **e** Heatmap displaying large-scale chromosomal Copy Number Variation levels inferred using InferCNV across the 12 epithelial subpopulations relative to non-malignant reference cells. Subpopulations with high CNV scores are classified as malignant tumor cells. UMAP Uniform Manifold Approximation and Projection, TNBC Triple-Negative Breast Cancer; Hormone Receptor-positive/HER2-negative breast cancer, HR + HER2- BC; CAF Cancer-Associated Fibroblasts.

To further dissect the functional programming of the identified CAF subtypes, particularly the TNBC-enriched my_iCAF population, a quantitative gene set scoring approach using UCell was implemented. This analysis evaluated the activity levels of curated hallmark gene sets relevant to CAF biology across the different subtypes. The results demonstrated a significant enrichment of several key signaling pathways within the my_iCAF subpopulation compared to the other CAF subtypes. Specifically, my_iCAFs exhibited markedly higher activity scores for the Transforming Growth Factor-beta (TGF_BETA_SIGNALING), Epithelial-Mesenchymal Transition (EPITHELIAL_MESENCHYMAL_TRANSITION), and PI3K-AKT-MTOR Signaling (PI3K_AKT_MTOR_SIGNALING) pathways (Fig. 3a, b). This finding provides additional molecular evidence corroborating the multifaceted nature of my_iCAFs, strongly suggesting their involvement in processes driving tumor progression, including cellular proliferation, survival, invasion, and metastasis, alongside their previously noted potential pro-inflammatory characteristics. Complementary to pathway activity analysis, the underlying TF regulatory networks governing the distinct phenotypes of the CAF subtypes were investigated. The analysis revealed that the regulatory landscape of my_iCAFs is markedly distinct from that of iCAFs, myCAFs, and dCAFs. Specifically, my_iCAFs displayed significantly higher inferred activity or expression levels of a unique set of TFs. Among the most prominently active TFs defining the my_iCAF state were *HDX*, *SRF*, *ETV1, CREB3L4*, and *TEAD1* (Fig. 3c). The identification of these specific TFs provides critical insights into the upstream regulatory mechanisms.

### Identification of tumor epithelial cells

Given the observed enrichment of the functionally distinct my_iCAF subpopulation within TNBC and its potential role in promoting tumor progression, subsequent analyses aimed to investigate the specific intercellular communication patterns between my_iCAFs and malignant epithelial cells, particularly focusing on differences between TNBC and HR + HER2- BC. To facilitate this interaction analysis, epithelial cells were extracted. Through an optimized single-cell analytical pipeline incorporating re-normalization, dimensionality reduction, and clustering, we identified 12distinct epithelial subclusters (Fig. 3d). To rigorously ascertain the malignant status of these identified epithelial subclusters, CNV analysis was performed. Significantly elevated levels of inferred chromosomal aberrations, a hallmark of malignancy, were detected in ten out of the twelve clusters. Conversely, two subpopulations, designated Tumor Epi9 and Tumor Epi12, exhibited comparatively fewer CNV events, suggesting they might represent non-malignant epithelial cells (Fig. 3e). Based on these CNV profiles, the subclusters displaying high levels of genomic alterations were confidently classified as malignant tumor epithelial cells, thereby providing validated tumor cell populations suitable for subsequent detailed investigation of their interactions with CAF subtypes.

### Aberrant intercellular signaling networks between my_iCAF and tumor cells in TNBC

**Fig. 4:**
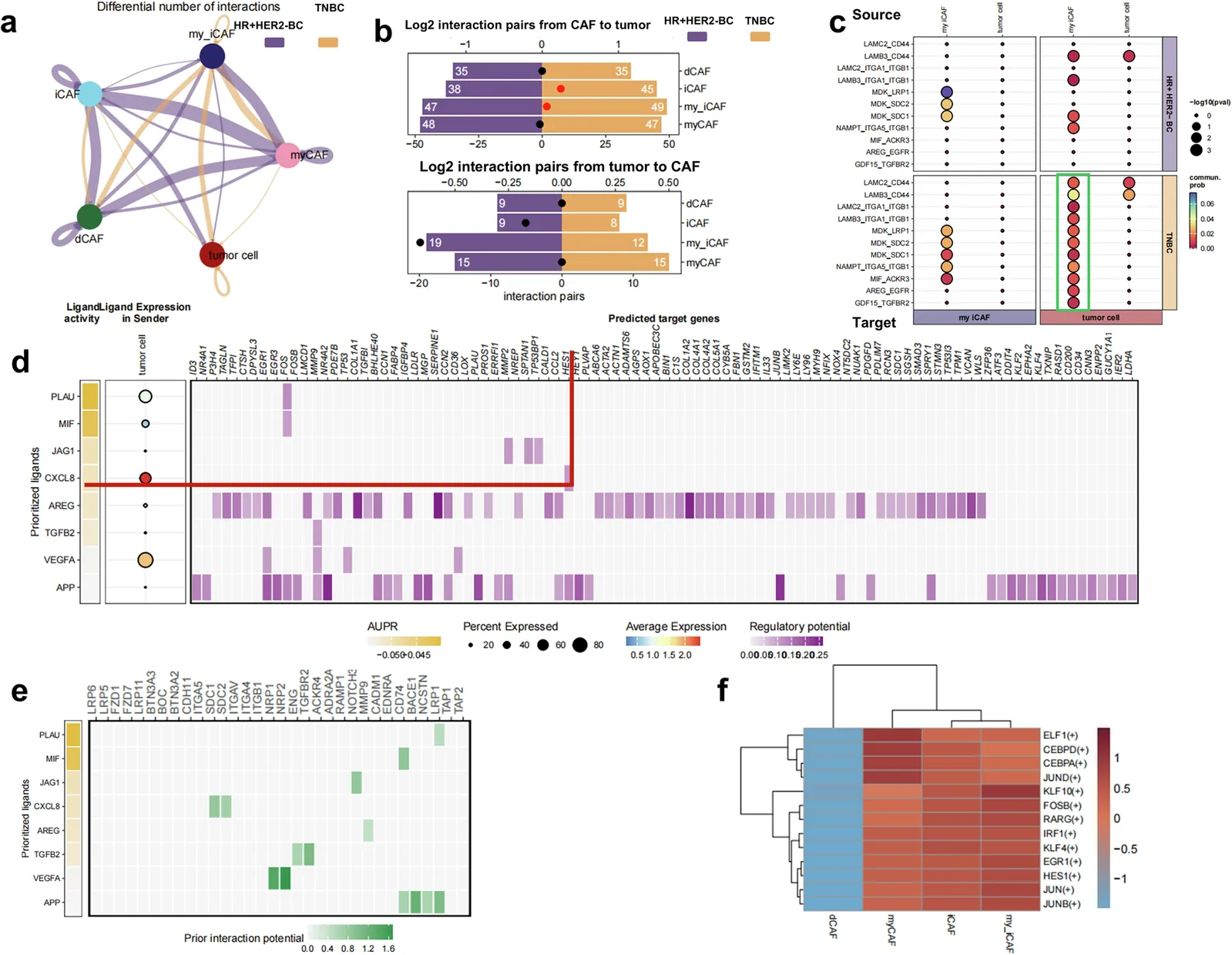
Intercellular Communication Networks Implicating my_iCAFs in the TNBC Microenvironment. **a** Circle plot summarizing the strength and number of inferred ligand-receptor interactions between CAF subtypes and tumor epithelial cells in TNBC versus HR + HER2- BC. **b** Bar chart quantifying the total number of significant interactions originating from each CAF subtype and targeting tumor cells, comparing TNBC and HR + HER2- BC microenvironments. **c** Bubble plot visualizing significant signaling pathways identified by CellChat as originating from my_iCAFs and targeting TNBC tumor cells. Key ligand-receptor pairs within pathways implicated in tumor progression (e.g., *LAMC2*-integrin, *MDK*-*SDC1*, *AREG*-*EGFR*, *GDF15*-*TGFBR2*) are highlighted. **d** NicheNet analysis predicting prioritized ligands expressed by TNBC or HR + HER2- BC tumor cells that potentially regulate target gene expression in my_iCAFs. Bubble plot showing TNBC-enriched ligands (e.g., *CXCL8*, *VEGFA*) and their predicted target genes, with *CXCL8* exhibiting the strongest TNBC-specific expression and targeting *HES1* in my_iCAFs. **e** Schematic details the inferred ligand-receptor pair. **f** Heatmap plot depicting the normalized expression or inferred activity of TFs predicted to regulate HES1 (e.g., *KLF10*, *FOSB*) across the four CAF subtypes. TNBC Triple-Negative Breast Cancer; Hormone Receptor-positive/HER2-negative breast cancer, HR + HER2- BC; CAF Cancer-Associated Fibroblasts.

To elucidate the potential functional consequences of the altered stromal composition observed in TNBC, a systematic analysis of intercellular communication networks was performed using CellChat. A striking difference emerged when comparing TNBC and HR + HER2- BC: the overall number and strength of predicted interactions originating from the my_iCAF/iCAF and targeting tumor cells were significantly elevated in the TNBC compared to the HR + HER2- BC (Fig. 4a). A global overview of the communication patterns further substantiated this finding, revealing that while various CAF subtypes engage in signaling crosstalk with tumor cells, the communication output specifically emanating from my_iCAFs towards tumor cells was markedly more pronounced within the TNBC microenvironment (Fig. 4b). Delving deeper into the specific signaling pathways mediating this intensified crosstalk, the analysis identified several communication signals sent from my_iCAFs to TNBC tumor cells. Key signals included *LAMC2* interacting with *ITGA1* and *ITGB1* (involved in cell adhesion and migration), *MDK* signaling through *SDC1* (implicated in growth and angiogenesis), *AREG* activation of *EGFR* (a critical driver of proliferation and survival), and *GDF15* potentially signaling via *TGFBR2* (associated with diverse roles including inflammation and cachexia) (Fig. 4c). These findings collectively suggest that the TNBC-enriched my_iCAF population utilizes a diverse repertoire of signaling molecules to engage in extensive communication with malignant cells, potentially orchestrating a microenvironment conducive to enhanced tumor growth, survival, invasion, and metastatic dissemination characteristic of the aggressive nature of TNBC.

### CXCL8 signaling from TNBC reprograms my_iCAFs toward a pro-tumorigenic state

Recognizing the significant enrichment of my_iCAFs within the TNBC microenvironment and their heightened interaction with tumor cells, further investigation focused on identifying signals originating from tumor cells that might contribute to the establishment and function of this specific CAF subtype. The NicheNet analytical framework was employed to predict ligand-receptor interactions where TNBC or HR + HER2- BC as the sending population and my_iCAFs serve as the receiving population, prioritizing interactions likely to influence gene expression within the my_iCAFs. Initial analysis across both tumor types identified several ligands with high predicted activity, including *PLAU*, *MIF*, *JAG1*, and *CXCL8*. However, differential analysis specifically comparing TNBC tumor cells to HR + HER2- tumor cells revealed that the ligands *CXCL8* and *VEGFA* were among the most upregulated and active signals emanating specifically from TNBC tumor cells towards my_iCAFs. This observation strongly suggests that TNBC cells actively secrete factors, notably *CXCL8* (a pro-inflammatory chemokine) and *VEGFA* (a key angiogenic factor), which may sculpt the my_iCAF phenotype towards pro-inflammatory and pro-angiogenic states (Fig. 4d). Focusing on the prominent *CXCL8* signal, which was markedly upregulated in TNBC tumor cells relative to HR + HER2- BC cells, its cognate receptors, *SDC1* and *SDC2*, were found to be expressed on CAFs. NicheNet predicted that the downstream target gene most likely regulated by this TNBC-specific *CXCL8* signaling axis in my_iCAFs is *HES1*, a transcriptional repressor involved in cell fate decisions (Fig. 4e). Elevated *HES1* expression in CAFs has been implicated in fostering a pro-tumorigenic microenvironment; *HES1*-high CAFs may secrete increased amounts of factors that stimulate tumor cell proliferation and survival, potentially through activation of signaling cascades like PI3K-AKT and MAPK pathways in the cancer. Furthermore, *HES1* activity might contribute to a feed-forward loop sustaining CAF activation and proliferation through the enhanced secretion of factors such as *PDGF* and TGF-β. To explore the potential transcriptional mechanisms linking the *CXCL8*-*SDC* receptor interaction to *HES1* upregulation, the pySCENIC workflow was utilized to identify TFs predicted to regulate *HES1* within the CAF population. This analysis revealed that specific TFs, including *KLF10* and notably *FOSB* (a component of the AP-1 complex often associated with inflammatory and stress responses), which are predicted regulators of *HES1*, exhibited higher inferred activity or expression levels specifically within the my_iCAF (Fig. 4f). These convergent findings delineate a potential mechanism whereby TNBC tumor cells, through elevated secretion of *CXCL8*, activate *SDC* receptors on my_iCAFs, leading to increased activity of TFs like *FOSB*, which in turn drives the upregulation of *HES1*, ultimately reinforcing a pro-tumorigenic and potentially pro-inflammatory state within the my_iCAF population.

### Trajectory modeling delineates fibroblast activation towards my_iCAF

**Fig. 5:**
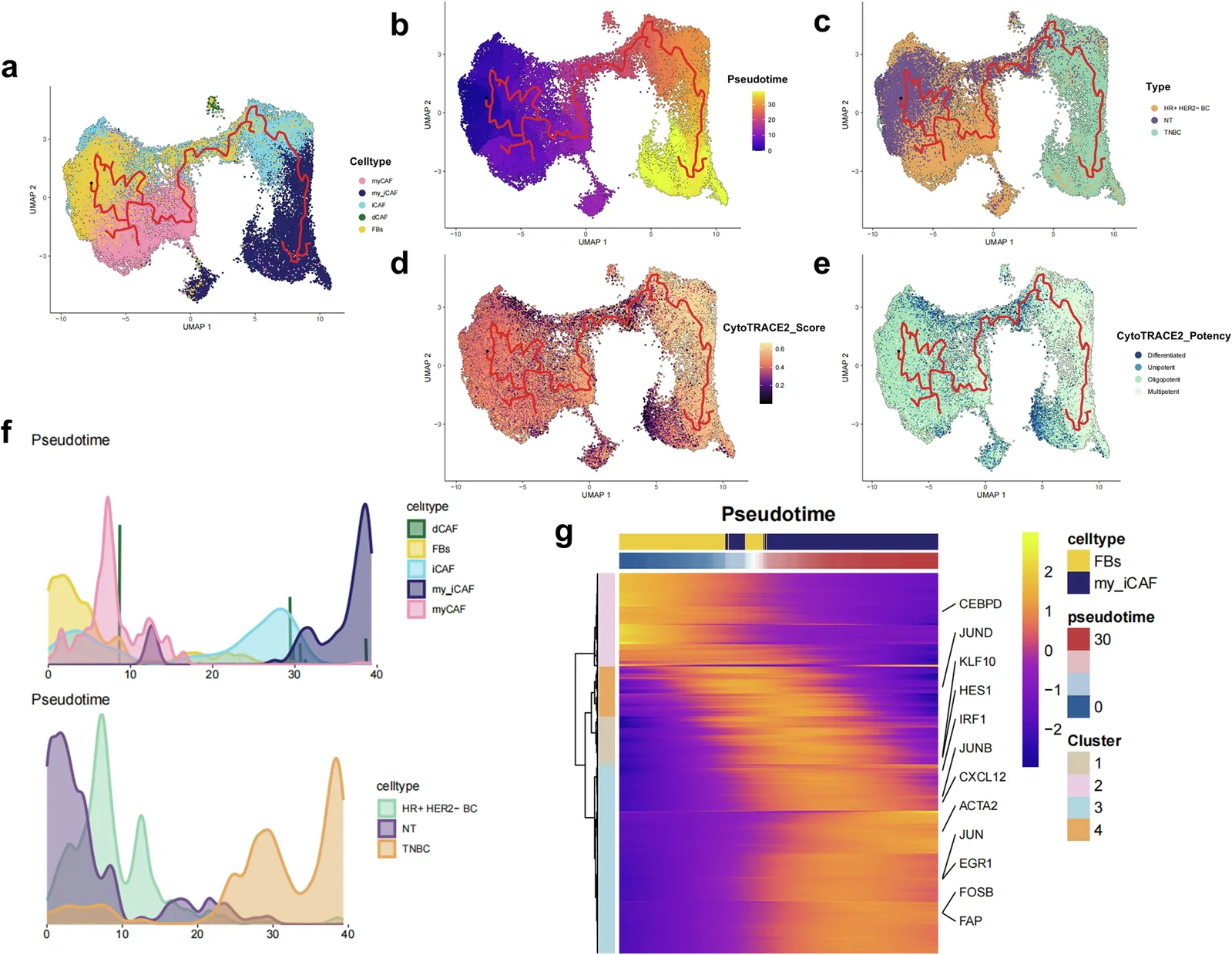
Pseudotemporal Trajectory Modeling Reveals Fibroblast Activation Towards a my_iCAF State. **a** UMAP embedding of normal fibroblasts and CAF subtypes, colored by Monocle3-inferred pseudotime, illustrating a trajectory from normal fibroblasts towards activated states. **b** Learned principal graph representing the differentiation trajectory overlaid onto the UMAP embedding. Root (normal fibroblasts) and terminal states (enriched in my_iCAFs) are indicated. **c** UMAP embedding colored by sample type (TNBC, HR + HER2- BC, NT), demonstrating the distribution of cells along the pseudotime trajectory relative to their origin. **d** UMAP embedding colored by scores reflecting cellular differentiation potential. **e** Visualization of predicted cell differentiation potential state by CytoTRACE2. **f** Density plot showing the relative abundance of each CAF subtype across pseudotime intervals, illustrating the shift towards the my_iCAF phenotype at later stages. **g** Heatmap depicting the dynamic expression profiles (smoothed expression along pseudotime) of representative my_iCAF marker genes and key transcription factors (e.g., *CXCL12*, *FAP*, *ACTA2*, *HES1*, *JUN*, *EGR1*, *FOSB*) during the inferred transition from normal fibroblasts to my_iCAFs. UMAP, Uniform Manifold Approximation and Projection; TNBC, Triple-Negative Breast Cancer; Hormone Receptor-positive/HER2-negative breast cancer, HR + HER2- BC; NT normal adjacent breast tissue, CAF Cancer-Associated Fibroblasts.

To reconstruct the potential developmental relationships and activation trajectories among fibroblast populations, single-cell trajectory inference was performed using Monocle3, integrating CAF subtypes identified via scRNA-seq with normal fibroblasts derived from NT. Normal fibroblasts were designated as the root state, representing the origin of the pseudotemporal continuum. The resulting trajectory analysis revealed a clear differentiation path, positioning the my_iCAF predominantly at the terminal end of the inferred developmental timeline. This finding suggests that my_iCAFs represent a late-stage, potentially terminally differentiated state within the spectrum of fibroblast activation observed in the breast tumor microenvironment (Fig. 5a–c). Intriguingly, analysis of CytoTRACE2 indicative of latent developmental potential suggested that my_iCAFs, despite their late pseudotemporal positioning, retain or exhibit heightened differentiation potential compared to intermediate states. This observation points towards a highly activated and plastic cellular state, capable of executing diverse CAF-associated functions critical for TME remodeling and potentially contributing to increased tumor heterogeneity (Fig. 5d, e). Examination of cell distribution along the pseudotime axis showed that normal fibroblasts were most abundant at the trajectory’s origin, gradually transitioning through intermediate states characterized by gene expression signatures partially overlapping with myCAFs and iCAFs, before ultimately converging towards the my_iCAF phenotype. Notably, CAFs originating from TNBC samples were significantly enriched in the later stages of this inferred trajectory compared to those from HR + HER2- BC, aligning with the observed enrichment of my_iCAFs in TNBC (Fig. 5f). To identify the molecular programs driving this transition, dynamic changes in gene expression were analyzed across the pseudotemporal trajectory leading from normal fibroblasts to my_iCAFs. This analysis confirmed the progressive upregulation of key my_iCAF marker genes, including *CXCL12*, *FAP*, and *ACTA2*, along the inferred differentiation path. Concurrently, specific transcription factors previously implicated in regulating *HES1* and potentially contributing to the my_iCAF phenotype, such as *HES1* itself along with *JUN*, *EGR1*, and *FOSB*, also exhibited significantly increased expression towards the terminal my_iCAF state. This coordinated upregulation of structural markers and regulatory factors along pseudotime potentially highlights the critical role of transcription factors like *JUN*, *JUNB*, *FOSB*, and *EGR1* in orchestrating the developmental program that culminates in the formation of the pro-tumorigenic my_iCAF subpopulation (Fig. 5g).

### ScATAC-seq reveals a distinct epigenome in my_iCAF

**Fig. 6:**
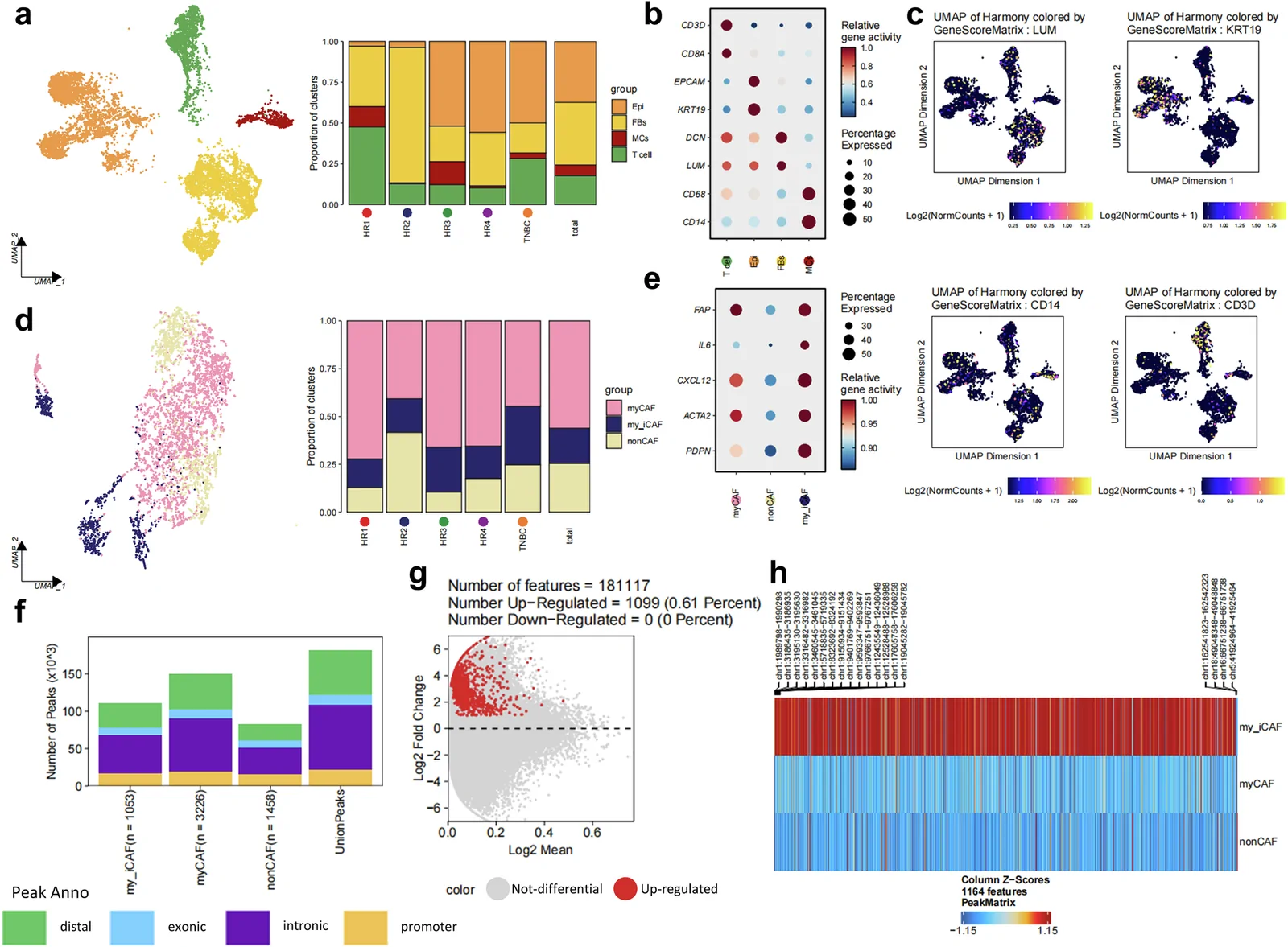
Epigenomic Profiling via Single-Cell ATAC Sequencing. **a** UMAP embedding of 14,958 high-quality cells profiled by scATAC-seq, color-coded by major cell types annotated based on gene activity scores. Inset shows cellular proportions per sample. **b** Dot plots displaying gene activity scores for canonical marker genes across major cell lineages identified by scATAC-seq. **c** UMAP feature plots showing the distribution of gene activity scores for selected marker genes. **d** UMAP embedding of reclustered fibroblasts based on scATAC-seq profiles, identifying epigenomically distinct CAF subtypes (e.g., my_iCAF, myCAF, nonCAF). Inset shows cellular proportions per sample for these subtypes. **e** Dot plots showing gene activity scores for CAF marker genes across the scATAC-defined fibroblast subtypes. **f** Bar chart illustrating the genomic distribution of accessible chromatin peaks relative to gene features (promoter, exon, intron, distal intergenic) for each CAF subtype. **g** Volcano plot highlighting differentially accessible chromatin regions identified when comparing my_iCAFs to other fibroblast subtypes. Significantly gained accessible regions in my_iCAFs are indicated. **h** Heatmap displaying normalized chromatin accessibility signals for the top significant differentially accessible chromatin regions enriched in the my_iCAF subtype across all analyzed fibroblast. Uniform Manifold Approximation and Projection; CAF Cancer-Associated Fibroblasts.

To complement the transcriptomic analysis and investigate the epigenetic regulatory layer contributing to fibroblast heterogeneity, scATAC-seq was performed. Following rigorous quality control filtering, a total of 14,958 cells yielded high-quality chromatin accessibility profiles suitable for downstream analysis. Initial computational processing, including data normalization, dimensionality reduction, and unsupervised clustering based on genome-wide chromatin accessibility patterns, successfully identified the major cell lineages present in the samples, including epithelial cells, fibroblasts, myeloid cells, and T cells. The overall epigenomic landscape and the relative contributions of each sample to the cell populations were visualized using UMAP (Fig. 6a). Cell type identities were further confirmed by calculating gene activity scores, which infer gene expression levels from chromatin accessibility signals in promoter and gene body regions; visualization of these scores for canonical marker genes demonstrated distinct patterns corresponding to the annotated cell types (Fig. 6b, c). Analogous to the scRNA-seq workflow, fibroblasts identified in the scATAC-seq data were computationally isolated for a more detailed sub-clustering analysis focused specifically on this lineage. This refined analysis of the fibroblast chromatin accessibility data revealed distinct epigenomic subtypes. Based on differential accessibility patterns at loci corresponding to key marker genes identified in the transcriptomic analysis (such as *FAP*, *ACTA2*, *CXCL12*), populations analogous to my_iCAFs and myCAFs were delineated, alongside a potential relative normal fibroblast state (‘nonCAF’) characterized by lower accessibility at these marker gene loci. The distribution of these epigenomically defined CAF subtypes was visualized via UMAP, and their relative proportions across samples were assessed (Fig. 6d). Visualization of gene scores for key CAF markers confirmed differential chromatin accessibility at these specific loci across the identified fibroblast subtypes (Fig. 6e). To identify subtype-specific regulatory elements, peak calling was performed using MACS2 for each CAF subtype, generating catalogues of accessible chromatin regions. The genomic distribution of these peaks indicated enrichment in expected regulatory regions, including promoters and intronic/intergenic enhancers (Fig. 6f). Crucially, differential accessibility analysis was conducted comparing the my_iCAF subtype to the other fibroblast populations. This revealed a significant number of genomic regions exhibiting heightened accessibility specifically in my_iCAFs, with 1,099 peaks showing statistically significant gains (FDR < = 0.1 & Log2FC > = 1). Notably, no significant decrease in peak accessibility was observed, suggesting a targeted opening of chromatin at specific regulatory loci characterizes the my_iCAF state rather than widespread chromatin condensation (Fig. 6g). A heatmap representation provided a comprehensive overview of these DARs that distinguish the epigenomic profile of my_iCAFs (Fig. 6h), setting the stage for investigating the transcription factors potentially binding these regions and regulating the my_iCAF phenotype.

### Enhanced chromatin accessibility at AP-1 transcription factor binding sites characterizes the my_iCAF epigenome

**Fig. 7:**
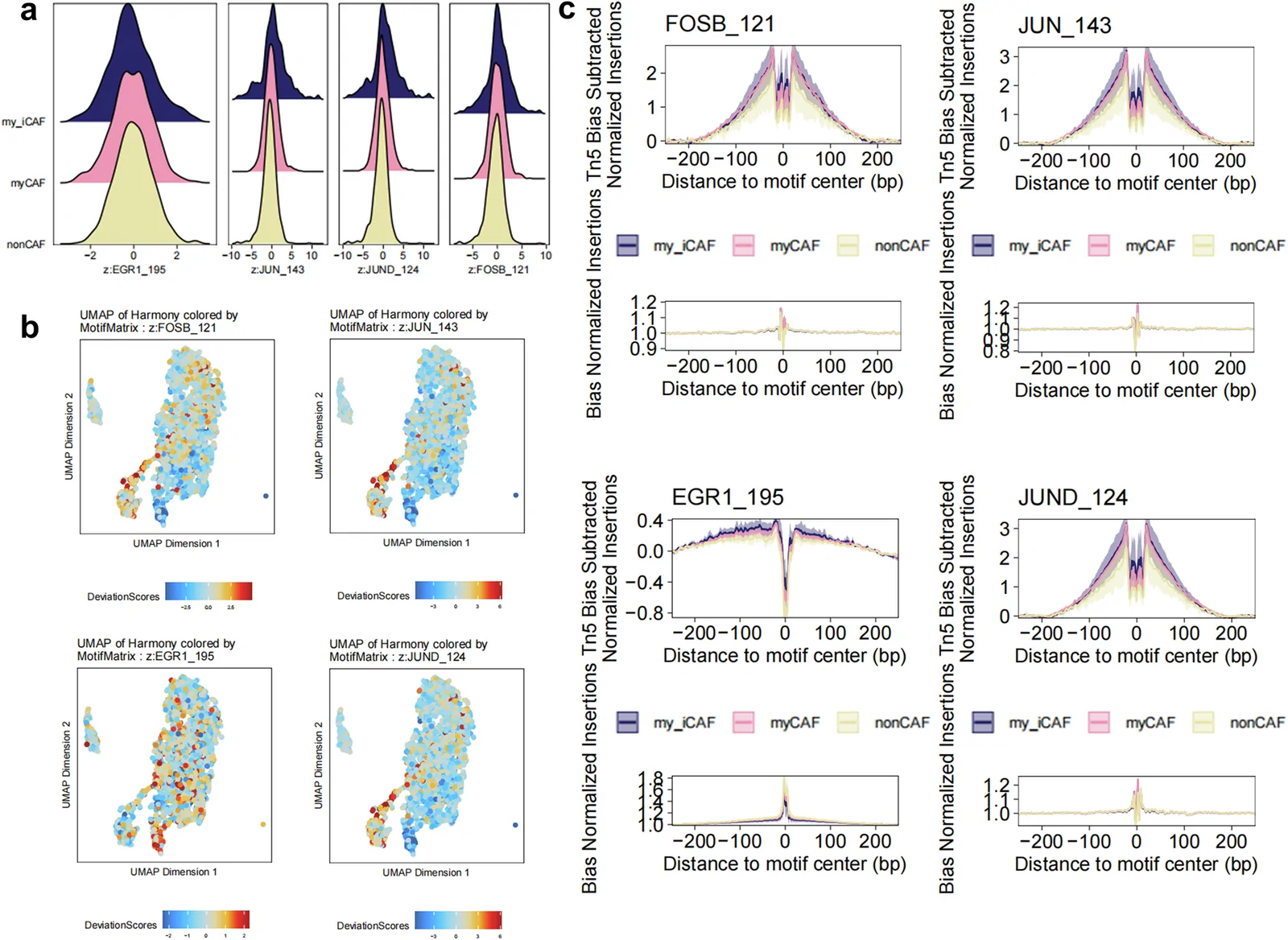
Transcription Factor Motif Accessibility and Occupancy Analysis. **a** Mountain landscape diagram showing enrichment scores (e.g., ChromVAR deviation scores) for DNA binding motifs of key transcription factors (*EGR1*, *JUN*, *JUNB*, *FOSB*) across scATAC-defined CAF subtypes. **b** UMAP embeddings overlaid with motif accessibility scores, visualizing the accessibility patterns for *EGR1*, *JUN*, *JUNB*, and *FOSB* motifs across the fibroblast landscape. Co-enrichment within the my_iCAF cluster is highlighted for AP-1 factors. **c** Aggregate footprinting plots showing Tn5 transposase insertion frequencies centered around the binding motifs for *JUN*, *JUNB*, and *FOSB*, specifically within the my_iCAF population. Characteristic depletion patterns flanked by increased accessibility provide evidence of TF occupancy.

Building upon the identification of DARs distinguishing the my_iCAF via scATAC-seq, further analysis focused on determining whether these regions were enriched for binding motifs of specific TFs implicated in regulating the my_iCAF phenotype. Building upon our prior transcriptomic and trajectory analyses that implicated key regulators (*FOSB*, *JUN*, *JUNB*, *EGR1*) in my_iCAF development, we systematically quantified the accessibility of their canonical binding motifs across epigenomically defined CAF subtypes. This comparative motif enrichment analysis revealed significantly higher chromatin accessibility scores specifically for motifs recognized by the AP-1 family members *FOSB*, *JUN*, and *JUNB* within the my_iCAF population compared to the myCAF subtype (Fig. 7a). This finding suggests that the regulatory elements controlling AP-1 target genes are disproportionately located within open, accessible chromatin regions in my_iCAFs. The distribution of motif accessibility further corroborated this observation; visualization on the UMAP embedding demonstrated that genomic regions containing *FOSB*, *JUN*, and *JUNB* motifs exhibited markedly elevated accessibility that co-localized predominantly with the my_iCAF cluster, displaying highly consistent patterns among these functionally related TFs (Fig. 7b). To gain evidence for the actual occupancy of these TFs at their predicted binding sites, motif footprinting analysis was performed. This technique examines the pattern of Tn5 transposase cleavage frequency surrounding a motif, where bound factors protect the DNA from cleavage, creating a characteristic depletion signal (the footprint) flanked by hypersensitive sites. The analysis revealed distinct, well-defined footprint patterns, characterized by the canonical “dip-peak” shape expected for stable TF binding, specifically at *FOSB*, *JUN*, and *JUNB* motifs within the my_iCAF. The presence of these archetypal footprints provides strong inferential evidence for the specific and stable binding of these AP-1 factors to their target sequences within the accessible chromatin landscape characteristic of my_iCAFs, thereby implicating AP-1 signaling pathways in establishing and maintaining the unique epigenomic and transcriptomic state of this TNBC-associated CAF subtype.

### Multi-Omic Integration Confirms FOSB as a Key Regulator Sustained by Epigenetic Mechanisms in my_iCAFs

**Fig. 8:**
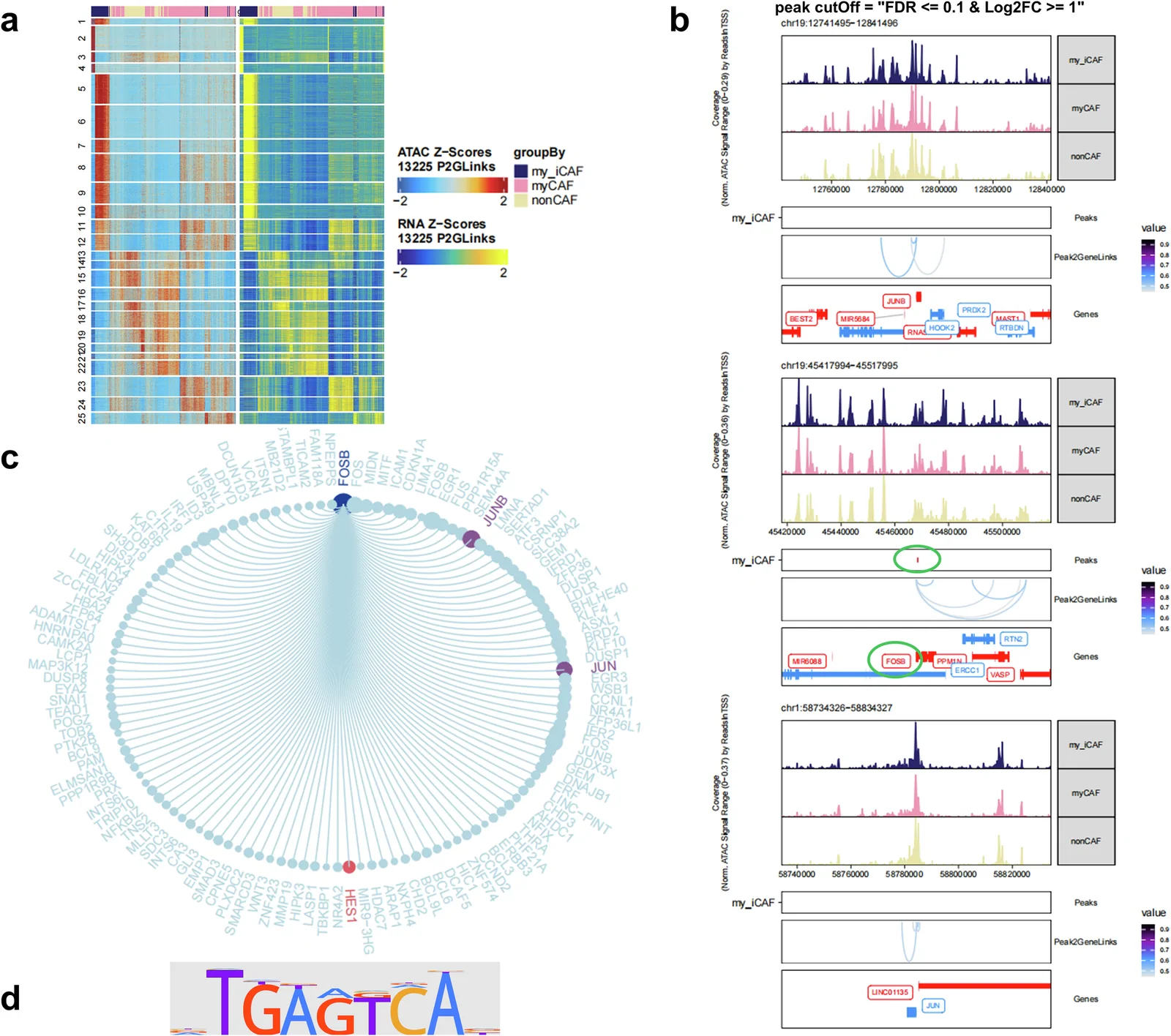
Integrated Multi-Omic Analysis Links Chromatin Accessibility to Gene Regulation. **a** Heatmap illustrating the correlation between chromatin accessibility of cis-regulatory elements (peaks) and the expression of linked genes, computed across single cells for each CAF subtype using an integrated scRNA/scATAC dataset. **b** Genome browser tracks displaying integrated data at representative loci (e.g., *FOSB*, *JUN*, *JUNB*). Tracks show scATAC-seq peaks, gene models, scRNA-seq expression coverage (separated by fibroblast subtype), and predicted peak-to-gene linkages. Positive correlation between *FOSB* locus accessibility and gene expression in my_iCAFs is highlighted. **c** Regulatory network diagram inferred using pySCENIC and multi-omic data, illustrating the predicted regulatory interactions centered around *FOSB*. Arrows indicate positive regulation of target genes including *HES1*, *JUN*, and *JUNB* by *FOSB* in my_iCAFs. **d** Sequence logo representing the canonical DNA binding motif for the *FOSB* transcription factor.

To establish a direct link between the observed epigenomic features and the transcriptomic profiles of CAF subtypes, particularly my_iCAFs, an integrative analysis correlating chromatin accessibility (from scATAC-seq) with gene expression (from scRNA-seq) at the single-cell level was conducted. Utilizing computational methods to link distal accessible chromatin peaks to putative target genes (e.g., via a GeneIntegrationMatrix approach), the overall relationship between chromatin openness and gene activity was assessed. A generally positive correlation was observed across the dataset, confirming the expected concordance between accessible regulatory elements and active gene transcription. Notably, this correlation between peak accessibility and linked gene expression appeared particularly strong within the my_iCAF relative to other CAF subtypes, suggesting a tightly coupled epigenomic and transcriptomic regulatory state in these cells (Fig. 8a). Focusing specifically on the regulatory landscape of my_iCAFs, the analysis correlated the accessibility of high-confidence DARs (filtered using criteria such as FDR ≤ 0.1 & Log2FC ≥ 1) with the expression of associated genes, paying particular attention to key TFs. Visualization using genomic track plots revealed a compelling relationship for *FOSB*: the chromatin regions surrounding the *FOSB* gene locus exhibited significantly increased accessibility specifically in my_iCAFs, and this heightened accessibility showed a strong positive correlation with *FOSB* mRNA expression levels within the same cells. In contrast, the correlations for *JUN* and *JUNB* were observed to be less pronounced. This specific finding strongly suggests that *FOSB* expression in my_iCAFs is maintained, at least in part, through positive feedback involving its own locus being epigenetically poised for transcription (Fig. 8b). Leveraging these integrated multi-omic insights, the regulatory network inference using pySCENIC was refined. The resulting network model centered on *FOSB* highlighted its potential role as a key upstream regulator within my_iCAFs. *FOSB* was predicted to exert positive regulatory control not only over *HES1*, as previously suggested, but also potentially over other AP-1 components, namely *JUN* and *JUNB*. This inferred network architecture, where *FOSB* influences both downstream targets and other AP-1 family members, aligns coherently with the scATAC-seq findings demonstrating co-localized enrichment of chromatin accessibility at *FOSB*, *JUN*, and *JUNB* motifs within the my_iCAF population (Fig. 8c).

### TNBC activates CAF subtype transition via the CXCL8/FOSB/HES1 axis

Collectively, our integrated analyses support a model wherein TNBC-secreted *CXCL8* activates *FOSB*, which then functions as a critical regulatory hub in my_iCAFs. This axis is potentially sustained through positive epigenetic self-regulation of *FOSB*, driving a downstream network that includes *HES1* and other AP-1 factors.

**Fig. 9:**
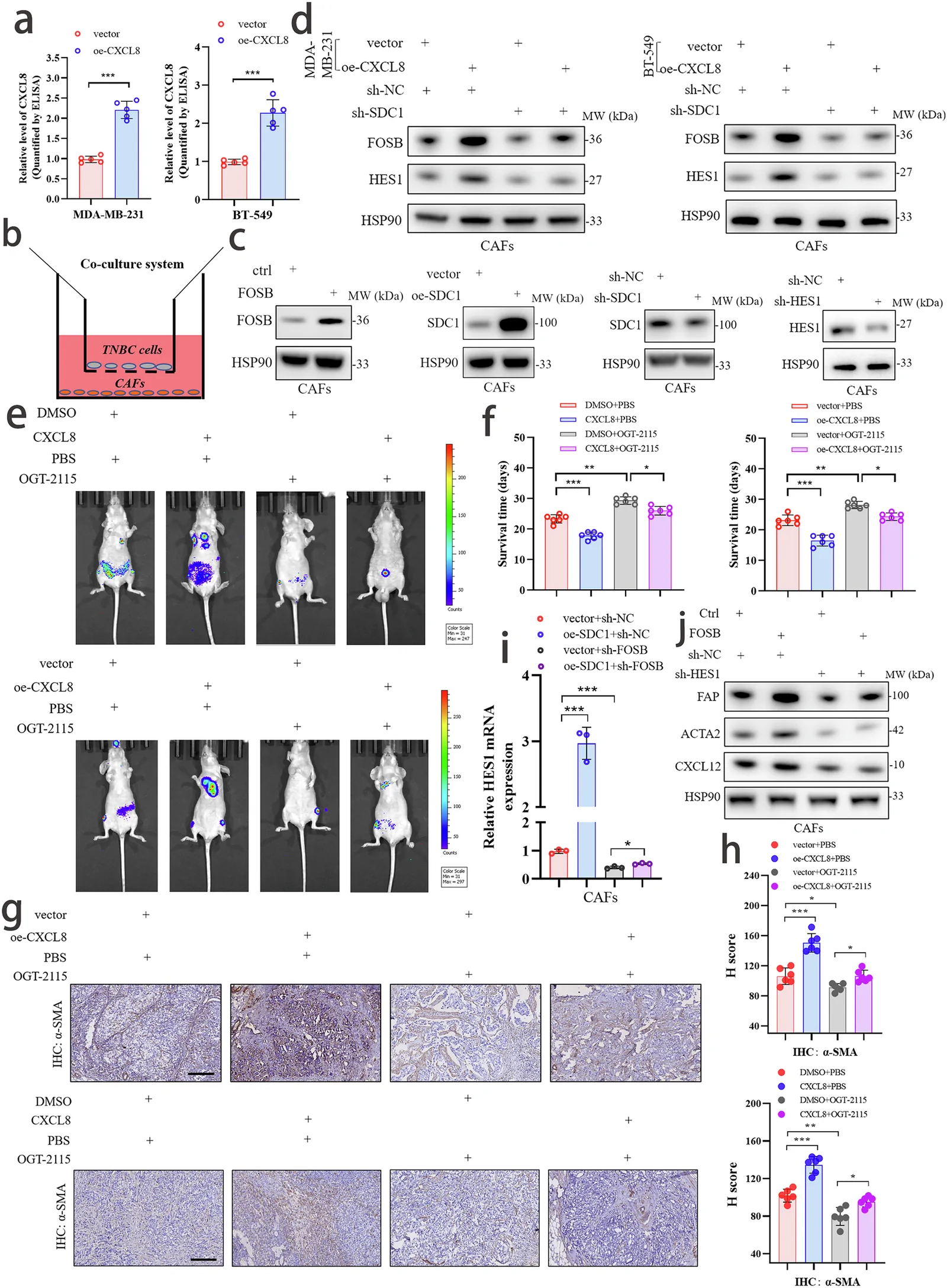
TNBC activates CAF subtype transition via the CXCL8/SDC1/HES1 axis. **a** ELISA analysis of CXCL8 secretion levels in different groups of TNBC cells. **b** Schematic diagram of co-culture between TNBC cells and CAFs. **c** Western blot analysis of overexpression efficiency (FOSB and SDC1) and knockdown efficiency (SDC1 and HES1). **d** Western blot analysis of FOSB and HES1 expression in CAFs in different experimental groups. **e**, **f** In vivo model of TNBC metastasis via tail vein injection showed metastatic potential in indicated treatment groups (**e**), survival time was recorded and analyzed (**f**). **g**, **h** The density of CAF cells in metastatic tumors of mice from different treatment groups was observed through α-SMA immunohistochemistry. **i** RT-qPCR analysis of HES1 mRNA expression levels across different treatment groups. **j** Western blot analysis of myCAF biomarker expression in indicated groups.

To investigate the potential relationship between TNBC and myCAFs, we first established TNBC cells overexpressing CXCL8 and examined the secretion of CXCL8 in TNBC cell line (Fig. 9a). Additionally, we cultured human-derived CAFs, and designed a tumor interaction study model (Fig. 9b). We obtained CAFs from TNBC tumors. The isolated CAFs grew well, and the cells showed typical fibroblast morphology (fusiform, fascicular, and scattered). We established stable cell lines overexpressing *FOSB/SDC1* or with *SDC1/HES1* knockdown (Fig. 9c). In the CAFs-TNBC co-culture system, SDC1 knockdown significantly reversed the activating effects of CXCL8 overexpression on the FOSB-HES1 signaling pathway in CAFs (Fig. 9d). In the in vivo TNBC metastasis model, we employed OGT2115 (a heparanase inhibitor [14]) to block SDC1 function. Our findings establish that SDC1 blockade effectively counteracts CXCL8-driven metastatic progression in TNBC models (Fig. 9e, f). Additionally, through pathological analysis of metastatic tumors in mice, we observed that overexpression of CXCL8 could drive the formation of more CAFs, while the use of OGT-2115 was able to reverse this trend (Fig. 9g, h). As a transcription factor, FOSB knockdown in CAFs significantly reversed the SDC1 overexpression-induced upregulation of HES1 mRNA (Fig. 9i). Also, we found that the FOSB-HES1 axis can effectively activate the transition of CAFs into my_iCAFs (Fig. 9j).

### My_iCAFs play an important role in the progression of TNBC

**Fig. 10:**
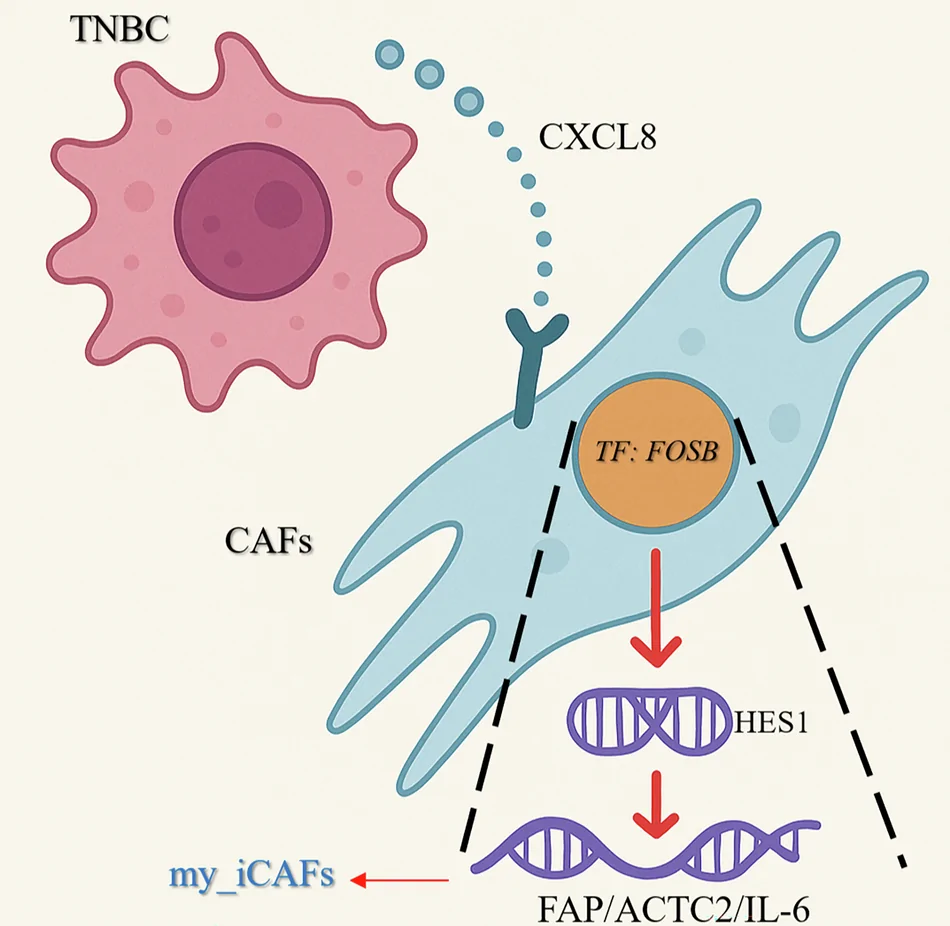
CXCL8 signaling activates the transition of CAFs into my_iCAFs via the FOSB-HES1 axis. Proposed mechanistic model of the study.

By collecting samples from breast cancer patients, we extracted and isolated NFs (Normal Fibroblasts) to induce my_iCAFs (Suppl. Fig. 1a, b). At the in vitro level, EdU assays observed that co-culturing my_iCAFs with TNBC significantly promoted the proliferation of TNBC (Suppl. Fig. 1c, d), and this conclusion was validated in vivo by mouse tumor formation experiments (Suppl. Fig. 1e, f). To further investigate the potential prognostic impact of my_iCAF, we performed deconvolution analysis on the TCGA-BRCA dataset using our scRNA-seq data as a reference, employing the “BayesPrism” algorithm (Suppl. Fig. 1g). To enhance accuracy, ribosomal and mitochondrial genes were filtered out during preprocessing. Protein-coding genes and long non-coding RNAs showed strong correlation between the single-cell and bulk RNA-seq datasets (Suppl. Fig. 1h), supporting the robustness of the deconvolution. Subsequent survival analysis revealed that patients with high my_iCAF abundance had a significantly worse prognosis [HR = 2.67, 95% CI: 1.93–4.62, *P* = 0.034] (Suppl. Fig. 1i). To elucidate the mechanism by which CXCL8-FOSB-HES1 induces my_iCAFs, we consulted the JASPAR database and obtained the potential binding motifs of FOSB (Suppl. Fig. 2a). At the mRNA level, we observed that knockdown of FOSB reversed the inducing effect of CXCL8 supplementation on HES1 mRNA expression (Suppl. Fig. 2b). ChIP assays confirmed that, within the predicted HES1 promoter sequence (the + 2000 bp to +500 bp region), the motif-containing site is a critical region for FOSB binding and regulation of HES1 (Suppl. Fig. 2c). Together, this CXCL8-FOSB-HES1 cascade orchestrates the distinct pro-tumorigenic transcriptional program characteristic of my_iCAFs, thereby reinforcing their pivotal role in shaping the aggressive TNBC microenvironment (Fig. 10).

## Discussion

The intricate interplay between tumor cells and their surrounding microenvironment is increasingly recognized as a critical determinant of cancer progression and therapeutic response, particularly in highly heterogeneous malignancies such as TNBC [7]. While the role of CAFs in shaping the TME is well-established [9], their functional diversity and subtype-specific contributions, especially when comparing distinct breast cancer subtypes like the aggressive TNBC and the more common HR + HER2- BC, have remained incompletely understood. Leveraging the power of integrated sc-RNA and sc-ATAC analyses, this study provides a high-resolution map of CAF heterogeneity within these distinct breast cancer contexts [11, 12]. Our central finding is the identification and comprehensive characterization of a novel CAF subpopulation, termed my_iCAF, which exhibits a unique molecular signature and is significantly enriched within the TNBC’s TME compared to HR + HER2- BC. This discovery, validated through in experimental analysis, highlights a previously underappreciated layer of stromal complexity specifically associated with TNBC [13].

The distinct phenotype of the TNBC-enriched my_iCAF population, characterized by the co-expression of markers typically associated with both myofibroblastic and inflammatory CAF subtypes, suggests a unique functional capacity tailored to the aggressive biology of TNBC. Classical myCAFs are primarily implicated in extracellular matrix deposition and remodeling, fostering invasion and stiffness, while iCAFs are known drivers of inflammation and immune modulation through cytokine secretion. The my_iCAF population identified herein appears to integrate these functions, potentially creating a uniquely supportive niche for TNBC progression. Pathway enrichment analyses corroborated this interpretation, revealing simultaneous enrichment of signaling pathways linked to epithelial-mesenchymal transition, PI3K/AKT/mTOR signaling crucial for proliferation and survival, alongside potent pro-inflammatory pathways such as TNF signaling via NF-κB and growth hormone signaling implicated in diverse tumorigenic processes. This convergence of functions within a single CAF subtype contrasts with more dichotomous models of CAF activity and suggests that my_iCAFs may synergistically promote multiple hallmarks of cancer—including invasion, proliferation, inflammation, and potentially immune evasion—thereby contributing significantly to the poor prognosis associated with TNBC [7].

Delving into the regulatory architecture underlying the distinct my_iCAF state, our integrated multi-omic analyses point towards specific transcriptional and epigenetic mechanisms. We identified a unique set of TFs, notably *FOSB*, exhibiting heightened activity within the my_iCAF population. *FOSB* often acts as part of the AP-1 complex, integrating diverse stimuli such as growth factors and inflammatory signals [15]. Consistent with the elevated TF activity, our scATAC-seq analysis revealed significantly increased chromatin accessibility at binding motifs for *FOSB* and related AP-1 factors specifically within my_iCAFs, supported by motif footprinting evidence suggesting direct factor occupancy. This distinct epigenetic landscape, characterized by accessible regulatory elements linked to the my_iCAF gene expression program, appears crucial for establishing and maintaining this unique cellular state. Furthermore, the observed positive correlation between *FOSB* gene expression and chromatin accessibility at its own locus suggests a potential positive autoregulatory feedback loop, which could contribute to the stability and persistence of the FOSB-high my_iCAF phenotype within the TNBC TME.

Beyond cell-intrinsic regulation, the dense network of intercellular communication within the TME plays a pivotal role. Our analyses revealed significantly enhanced communication signals involving my_iCAFs within the TNBC context compared to HR + HER2- BC, as inferred through ligand-receptor interaction analyses. Specifically, my_iCAFs were predicted to signal to tumor cells via pathways known to promote proliferation and survival, such as *AREG*–*EGFR* signaling. Perhaps more revealingly, we identified evidence of signaling directed from TNBC tumor cells towards my_iCAFs, potentially shaping their phenotype. The predicted interaction involving elevated TNBC-derived *CXCL8* signaling through *SDC* receptors on CAFs, leading to the downstream activation of the *FOSB/HES1* axis, provides a compelling molecular mechanism by which TNBC cells might actively “educate” surrounding fibroblasts, promoting the differentiation or maintenance of the pro-tumorigenic my_iCAF population. This highlights a dynamic, bidirectional crosstalk wherein tumor cells and specific CAF subtypes co-evolve, creating a microenvironment that sustains malignant progression, particularly in the aggressive setting of TNBC.

The distinct enrichment and multifaceted pro-tumorigenic profile of the my_iCAF subpopulation within TNBC carry significant potential clinical implications. Given TNBC’s inherent aggressiveness and limited targeted therapeutic options, understanding the specific TME components that support its growth and metastasis is of paramount importance [7]. Our findings strongly suggest that the my_iCAF population may function as a key stromal driver contributing to the poor prognosis commonly associated with TNBC. Furthermore, the detailed molecular characterization of my_iCAFs opens avenues for therapeutic exploration. Targeting this specific CAF population directly, or interfering with the key regulatory nodes (e.g., *FOSB* activity) or signaling pathways (e.g., the *CXCL8*–*SDC* axis, *AREG*–*EGFR* signaling) crucial for their maintenance and function, might represent a viable strategy to selectively disrupt the supportive TNBC TME and potentially sensitize tumors to existing therapies. It is important to acknowledge that the current study primarily establishes correlative relationships among CXCL8, FOSB, and HES1 in TNBC progression. Although our data reveal consistent associations between the expression levels of these factors and aggressive tumor phenotypes, and we have also observed certain regulatory interactions. The proposed pathway should therefore be interpreted as a candidate mechanistic hypothesis derived from our correlative findings, requiring further experimental validation.

While this study provides valuable insights through its integrated multi-omic single-cell approach and in situ validation, certain limitations should be acknowledged. Our analyses relied on publicly available datasets, which, despite rigorous batch correction, may retain residual technical variations and often lack comprehensive clinical outcome data, precluding direct correlation between CAF subtypes and patient survival in this study. Additionally, while this work focused on CAFs, it did not comprehensively explore interactions with other critical TME components, such as diverse immune cell subsets or endothelial cells, which undoubtedly influence tumor progression and warrant further investigation [4]. Finally, the scope of the in situ validation was limited in terms of patient cohort sizes and the multiplexing capacity for simultaneously interrogating multiple markers. Future studies with larger patient cohorts and expanded biomarker panels will be essential to confirm and extend these findings toward clinical translation.

## Materials and Methods

### ScRNA-seq data processing

Bioinformatic processing of the sc-RNA data was conducted using the Seurat package (v5.0.1) in R (v4.3.0) [11]. Initial quality control involved filtering individual cells based on criteria below: number of detected genes per cell between 200 and 10,000; total Unique Molecular Identifier counts greater than 1,000; and percentage of mitochondrial gene counts less than 20%. Potential doublet cells within each sample were identified and removed using the filterDoublets function from the scDblFinder package [16]. Post-filtering, gene expression counts for each cell were normalized using the NormalizeData function employing the “LogNormalize” method with a scale factor of 10,000. The top 2000 highly variable genes were identified using the FindVariableFeatures function. To mitigate batch effects between datasets and samples, the Harmony algorithm (RunHarmony function) was applied to the principal component analysis space derived from the scaled expression values of highly variable genes [17]. The top 30 harmony-corrected principal components were used for downstream analyses, including graph-based clustering (FindNeighbors and FindClusters functions with an appropriate resolution parameter) and non-linear dimensionality reduction for visualization via Uniform Manifold Approximation and Projection (UMAP) (RunUMAP function).

Initial cell type annotation was performed by evaluating the expression of established canonical marker genes within the identified cell clusters. Key markers included *PECAM1*, *PTPRB*, and *PLVAP* for endothelial cells; CD79A for B cells; *CD3D* and *CD4* for CD4 + T cells; *CD3D*, *GZMK*, and *CD8A* for CD8 + T cells; *NKG7* and *GNLY* for NK cells; *CD68* and *CD14* for myeloid cells; *EPCAM* and *KRT19* for epithelial cells; *DCN* and *LUM* for fibroblasts; and *RGS5* for pericytes. Fibroblast cells were subsequently isolated for detailed subclustering analysis. Based on established literature definitions and marker expression, fibroblasts were categorized into distinct subpopulations: inflammatory CAFs (iCAFs; characterized by *IL6*, *CXCL12*), myofibroblastic CAFs (myCAFs; characterized by *FAP*, *ACTA2*, *PDPN* and *PDGFRB*), detached CAFs (dCAFs; characterized by *SCRG1*), and a mixed-phenotype my_iCAF exhibiting both inflammatory and myofibroblastic features [18]. Differential abundance of these CAF subtypes between TNBC and HR + HER2-BC was assessed using the Milo package [19], assigning cells to neighborhoods on a k-nearest neighbor graph (k = 30, prop = 0.2). Differentially expressed genes upregulated within key CAF subtypes (which was most enriched CAF type in TNBC) were identified using Seurat’s FindAllMarkers function (log2 (Fold Change) > 0.25, min.pct > 0.25, adjusted *p*-value < 0.05). Functional enrichment of these upregulated genes was investigated using the ClusterProfiler package for KEGG pathway analysis [20]. To evaluate overall pathway activity scores at the single-cell level, the UCell package was employed with curated CAF-relevant gene sets from the MSigDB Hallmark collection.

### Transcription factor (TF) analysis

TF regulatory networks (regulons) and their activity were constructed using the pySCENIC package (v0.12.1) on the normalized expression matrix [21]. The GRNboost2 algorithm was used for gene regulatory network reconstruction, and motifs from the cisTarget Human motif database (v9; motifs-v9-nr.hgnc-m0.001-o0.0.tbl) were utilized for motif annotation. Regulon activity in individual cells was quantified using the AUCell algorithm.

### The copy number variation (CNV) analysis

CNVs were computationally inferred for epithelial cell populations in both TNBC and HR + HER2- BC samples using infercnv package (v1.18.1) [22]. Epithelial cells from NT were designated as the reference group. Analysis was performed with denoising enabled (denoise = TRUE) and a cutoff of 0.1 to identify chromosomal alterations indicative of malignant status.

### Cellular communication analysis

Intercellular communication between CAF subtypes and tumor cells were investigated using two complementary approaches. The CellChat package (v1.6.1) [23] with its associated ligand-receptor database (CellChatDB.human) was employed to infer and compare overall communication patterns and strengths between TNBC and HR + HER2- contexts, utilizing the computeCommunProb and filterCommunication functions. Using NicheNet (v2.2.0), we systematically compared tumor cell (sender) to CAF (receiver) communication networks in TNBC versus HR + HER2- BC, applying ligand activity filters (expression_pct ≥ 0.05 in both populations) to identify subtype-specific ligand-receptor interactions [24].

### Bulk-RNA seq deconvolution and survival analysis

For bulk RNA-seq deconvolution, we employed the R package “BayesPrism” (version 2.2.2) 27, which offers improved accuracy in resolving tumor cell populations. The deconvolution was conducted using default parameters. To assess the association with clinical outcomes, univariate and multivariate Cox regression analyses for overall survival were carried out utilizing the “survival” (version 3.6-4) and “rms” (version 6.8-0) R packages.

### Trajectory analysis

Potential developmental relationships and differentiation trajectories among fibroblast populations were explored using the Monocle3 package (v1.3.1) [25]. The UMAP embedding derived from Seurat was used as the reduced dimensionality input for trajectory reconstruction. Fibroblasts originating from NT samples were defined as the trajectory root (pseudotime origin). Cellular differentiation potential along the trajectory was assessed using CytoTRACE2 [26]. Genes exhibiting significant expression changes along the inferred pseudotime trajectory (specifically comparing normal fibroblasts to the key TNBC-associated CAF state) were identified using the graph_test function, applying significance thresholds of *q*-value < 0.001 and Moran’s I > 0.25.

### ScATAC-seq and data processing

Bioinformatic processing of raw scATAC-seq data was performed primarily using the ArchR package (v1.0.3) [12]. Initial quality control involved filtering nuclei based on Transcription Start Site (TSS) enrichment scores (minimum TSS enrichment = 4) and the number of unique nuclear fragments (minimum 1,000 fragments). Potential doublets were identified using the addDoubletScores function and removed using filterDoublets. Dimensionality reduction was performed using Iterative Latent Semantic Indexing (LSI) on the tile matrix (addIterativeLSI function; parameters: iterations = 4, resolution = 4, varFeatures = 25000, dimsToUse = 1:30). Batch effects between samples were corrected using the Harmony algorithm implemented within ArchR (addHarmony). Cell clustering was performed on the Harmony-corrected LSI dimensions using the addClusters function, and results were visualized using UMAP (addUMAP). Chromatin accessibility peaks were called using MACS2 [27]. After generating pseudo-bulk replicates for cell groups using the addGroupCoverages function (minCells = 50) to enhance signal robustness.

Cell type annotation for the scATAC-seq data was performed using ArchR’s implementation of gene activity scores, calculated based on chromatin accessibility within gene bodies and promoter regions. These scores were used to identify major lineages via established marker genes (T cells: *CD3D*, *CD8A*; Epithelial cells: *EPCAM*, *KRT19*; Fibroblasts: *DCN*, *LUM*; Myeloid cells: *CD68*, *CD14*). Annotation refinement and CAF subtyping (iCAF: *IL6*, *CXCL12*; myCAF: *FAP*, *ACTA2*, *PDPN*; my_iCAF: exhibiting features of both) were achieved by integrating the scATAC-seq data with the annotated scRNA-seq dataset using ArchR’s addGeneIntegrationMatrix function, leveraging the higher resolution of the transcriptomic data for constrained label transfer. Differentially accessible chromatin regions (DAR) between specified cell groups (e.g., comparing my_iCAFs to other CAF subtypes) were identified using statistical tests implemented in ArchR, applying significance thresholds of False Discovery Rate ≤ 0.1 and Log2 Fold Change ≥ 1. Results were visualized using volcano plots and heatmaps.

### Motif footprinting analysis

TF motif analysis was conducted to identify TFs potentially driving subtype-specific accessibility patterns (addDeviations function). Furthermore, TF occupancy at specific binding sites was inferred using motif footprinting analysis, which examines Tn5 transposase insertion patterns around known motifs, correcting for inherent Tn5 insertion bias to reveal characteristic depletion signals indicative of factor binding [28].

### Integration of multi-omic single-cell data

To comprehensively investigate the interplay between chromatin accessibility and gene expression, the processed scATAC-seq and scRNA-seq datasets were integrated using functionalities within ArchR. Peak-to-gene linkages were predicted using the getPeak2GeneLinks function, which correlates chromatin accessibility of distal peaks and promoter regions with the expression of target genes across single cells within the integrated dataset. Significant linkages were defined based on stringent statistical criteria (Pearson correlation coefficient > 0.45 and adjusted *p*-value < 0.0001), and these high-confidence peak-gene pairs were visualized using plotPeak2GeneHeatmap. For detailed inspection of regulatory interactions at specific loci of interest (e.g., key TFs), the plotBrowserTrack function was utilized to display scATAC-seq signal, peak calls, gene annotations, significant peak-to-gene links, and optionally scRNA-seq coverage tracks. Additionally, potential coordination between regulatory elements was explored by identifying significantly co-accessible peak pairs genome-wide using the addCoAccessibility function. Constrained integration via addGeneIntegrationMatrix also enabled direct comparison and correlation analyses between modalities at the single-cell level.

### Cell culture and reagents

The human triple-negative breast cancer (TNBC) cell lines MDA-MB-231 (RRID: CVCL_0062) and BT-549 (RRID: CVCL_1092) were obtained from the American Type Culture Collection (ATCC, Manassas, VA, USA) and cultured according to the provider’s protocols. Cell lines in this study were free of mycoplasma contamination. Prior to experimental use, all cell lines underwent mycoplasma testing with negative results and were authenticated through short tandem repeat (STR) analysis. Cell culture was performed using MEM medium (Gibco) containing 10% fetal bovine serum (FBS, Gibco), with incubation under standard conditions of 37 °C and 5% CO₂ in a humidified atmosphere. A non-contact co-culture system was established using 0.4 μm pore transwell chambers (Corning Costar, USA). CAFs (4 × 10⁵ cells/ml) were seeded in the lower compartment, while TNBC cells (2 × 10⁵ cells/ml) were plated in the upper chamber.

### Immunohistochemistry

Immunohistochemical staining of TNBC tumor tissue microarrays was performed using standard protocols. Briefly, paraffin-embedded sections were deparaffinized in xylene and rehydrated through a graded ethanol series. To block endogenous peroxidase activity, tissues were treated with 3% H₂O₂ for 15 min. Antigen retrieval was carried out by heating the samples in a retrieval buffer for 30 min. Primary antibody incubation (1:4000 dilution) was conducted overnight at 4 °C, followed by application of HRP-conjugated secondary antibodies. Signal visualization was achieved using DAB (3,3′-diaminobenzidine), and nuclei were counterstained with hematoxylin. Finally, slides were dehydrated in an alcohol-xylene series and mounted for analysis.

### Tail vein injection tumor model

For tumor cell inoculation, log-phase cultured cells (MDA-MB-231 and BT-549) were harvested, washed, and resuspended in ice-cold PBS at a concentration of 1 × 10⁶ cells/100 µL. Six-week-old mice (BALB/c, nude) were prepared by warming their tails at 37 °C for 1–2 min to dilate veins before being restrained in a specialized holder under sterile conditions. The injection procedure involved disinfecting tails with 70% ethanol, carefully inserting a 27–30 G needle into the lateral tail vein, and slowly administering 100 µL cell suspension over 30 s, followed by pressure application for hemostasis. Post-injection monitoring included acute distress observation during the first 30 min and weekly tumor engraftment evaluation through bioluminescence imaging (for luciferase-labeled cells).

### Bioluminescence Imaging

In vivo bioluminescence imaging was performed under isoflurane anesthesia. D-Luciferin was administered intravenously, and image acquisition commenced after a 1-minute delay. Signal intensity was analyzed using Living Image software (v4.4) with region-of-interest (ROI) quantification.

### Enzyme-Linked Immunosorbent Assay (ELISA)

CXCL8 levels in cell culture supernatants were measured using the Human CXCL8 ELISA Kit (EK108, Liankebio) with triplicate measurements. Following the manufacturer’s protocol, samples were incubated with detection antibody for 1 h at room temperature. After six washes with PBST, TMB substrate was added for colorimetric development (15 min). The reaction was stopped with 100 µL Terminate Solution, and absorbance readings were taken at 450 nm (primary) and 570 nm (reference).

### RNA Isolation and qRT-PCR

Total RNA was extracted with TRIzol reagent (Takara Bio Inc, #9108) according to the manufacturer’s protocol. cDNA synthesis was performed using 1 μg RNA with the Hiscript® III Reverse Transcriptase kit (Vazyme, #R223-01). Quantitative PCR analysis was carried out on a QuantStudio™ 3 system with ChamQ Universal SYBR qPCR Master Mix (Vazyme, #Q711-02). Gene expression levels were normalized to GAPDH and calculated using the 2 − ΔΔCT method.

### Protein extraction and western blot

Protein lysates were prepared from cultured TNBC cells or CAFs using RIPA buffer (Cell Signaling) containing 1 mM PMSF (Beyotime) and 1× protease/phosphatase inhibitors (Roche). Protein concentration was determined by BCA assay (Beyotime), with 30 μg aliquots separated on 4–20% SDS-PAGE gels.

For immunoblotting, proteins were transferred to 0.22 μm PVDF membranes (Roche) using a Trans-Blot Turbo system (Bio-Rad). Membranes were blocked with 5% non-fat milk in TBS-T for 3 h at 37 °C, then incubated overnight at 4 °C with primary antibodies (diluted in Beyotime buffer). After TBS-T washes, membranes were probed with HRP-conjugated secondary antibodies (1% milk/TBS-T) for 1 h at room temperature. Protein bands were detected using an ECL system (Millipore).

### Cancer-Associated Fibroblast (CAF) Isolation and Culture

Primary CAFs were obtained from tumor tissues through enzymatic digestion using the Human Tumor Dissociation Kit (Miltenyi, Germany). Following mechanical disruption, single-cell suspensions were generated, and fibroblasts were isolated based on their differential adhesion properties compared to epithelial cells. After trypsinization and homogenization, the purified CAFs were expanded in DMEM/F12 medium supplemented with 10% exosome-depleted FBS (Systembio, USA), penicillin (100 U/ml), and streptomycin (100 μg/ml), maintaining standard culture conditions (37 °C, 5% CO_2_) for 2–3 passages before experimentation.

### Subcutaneous xenograft tumor model

Athymic nude mice (BALB/c-nu/nu, 4–6 weeks old, body weight 18–22 g) were maintained under specific pathogen-free (SPF) conditions with a controlled temperature (22–25 °C), and humidity (50–60%). TNBC cells (MDA-MB-231) in the logarithmic growth phase were harvested by trypsinization (0.25% trypsin-EDTA), washed twice with cold phosphate-buffered saline (PBS), and resuspended in serum-free medium or PBS. For co-inoculation experiments, my_iCAFs were mixed with TNBC cells at a defined ratio (1:1). The cell suspension was mixed with an equal volume of Matrigel (Corning, #356234) to a final concentration of [e.g., 5 × 10⁶ cells/100 μL] and kept on ice. Each mouse received a subcutaneous injection of 100 μL of the cell suspension into the right or left flank using a 26-gauge needle. Prior to injection, the injection site was sterilized with 75% ethanol. Tumor growth was monitored every 2–3 days. Tumor length (L) and width (W) were measured using a digital caliper. Tumor volume (V) was calculated according to the formula:V = (L × W²)/2.

### EdU assay

Cells were seeded into 96‑well plates at 5 × 10³ cells/well and cultured overnight. EdU (RiboBio) was added to the medium at a final concentration of 10 μM and incubated for 2 h at 37 °C. Cells were then fixed with 4% paraformaldehyde, permeabilized with 0.5% Triton X‑100, and subjected to Click reaction using a Click‑iT™ EdU imaging kit (Invitrogen) according to the manufacturer’s protocol. Nuclei were counterstained with Hoechst 33342 (1 μg/mL). Fluorescence images were acquired using a fluorescence microscope, and the percentage of EdU‑positive cells was calculated.

### Chromatin immunoprecipitation (ChIP) assays

Chromatin immunoprecipitation (ChIP) was performed following the manufacturer’s protocol (EZ-Magna ChIP A/G kit, Millipore, 17-10086). Enrichment of FOSB at the HES1 promoter region was assessed using specific antibodies, with normal mouse IgG as the negative control. Precipitated DNA was subsequently analyzed by qPCR.

### Statistical analysis

Statistical analyses were performed using R software (v4.3.0; https://www.r-project.org/) and associated packages, or GraphPad Prism (v9.3; GraphPad Software). Comparisons of continuous variables between two independent experimental groups (e.g., TNBC vs. HR + HER2-) were typically conducted using the two-sided Wilcoxon rank-sum test, unless data distribution assumptions permitted the use of the two-sided Student’s t-test. Correlations between variables were assessed using either Pearson’s or Spearman’s rank correlation test, as appropriate for the data type and distribution. Statistical methods embedded within specific bioinformatics packages were used for differential expression (e.g., Wilcoxon test with Bonferroni correction in Seurat), differential accessibility, pathway enrichment analyses, and differential abundance testing, employing False Discovery Rate or adjusted *p*-value thresholds as described in the respective analysis sections. Throughout the study, a *p*-value or adjusted p-value threshold of less than 0.05 was considered statistically significant. Specific tests and significance levels are indicated in the figure legends or results text where applicable.

### Availability of supporting data

The primary single-cell RNA sequencing (scRNA-seq) and single-cell ATAC sequencing (scATAC-seq) datasets analyzed in this study are publicly available and were obtained from the National Center for Biotechnology Information (NCBI) Gene Expression Omnibus (GEO) database (https://www.ncbi.nlm.nih.gov/geo/). The specific accession numbers for the datasets used are GSE243526 and GSE279219. Further details regarding the original data can be found in the associated publications. Custom analysis scripts and code used for generating the results presented in this manuscript are available from the corresponding author upon reasonable request. The bulk RNA data were obtained from the TCGA-BRCA dataset.

## Supplementary information

Supplementary Table 1

Supplementary Figures

unedited blot

## Data Availability

This study utilized publicly available datasets deposited in the Gene Expression Omnibus (GEO) database (Table S1). Specifically, sc-RNA data were obtained from datasets GSE243526 and GSE279219 [29, 30]. The GSE243526 dataset comprised samples from 11 HR + HER2- BC patients, 4 individuals with normal adjacent breast tissue (NT), and 1 TNBC patient. The GSE279219 dataset exclusively contained samples from 13 TNBC patients. scATAC-seq data were derived from the GSE243526 dataset, with analysis focused on 4 selected HR + HER2- BC samples and 1 TNBC sample, based on quality control metrics and sample balance considerations. All data were generated and deposited by the original study investigators, who obtained informed consent from participants and requisite ethical approvals from their respective institutional review boards, in accordance with the Declaration of Helsinki. Tissue handling protocols appropriate for preserving single-cell viability and integrity for downstream sequencing applications were employed by the original data producers.
