# Supplementary material for: Single-cell multi-omics deciphers the myofibro-inflammatory program of cancer-associated fibroblasts in triple-negative breast cancer
Source: Cell Death Discov. 2026 Jul 11;12:404. doi: 10.1038/s41420-026-03255-z (PMC13642949; doi:10.1038/s41420-026-03255-z)
Supplement: Supplementary file 1 [file 41420_2026_3255_MOESM1_ESM.docx]

Table S1. Summary of the datasets.

| Sequencing type | Series | Sample size |
| --- | --- | --- |
| SC-RNA seq | GSE243526 (GEO) | 11 HR+HER2- BC, 4 NT, 1 TNBC |
| SC-RNA seq | GSE279219 (GEO) | 13 TNBC |
| SC-ATAC seq | GSE222703 (GEO) | 4 HR+HER2- BC, 1 TNBC |

SC-RNA seq, Single-Cell RNA sequencing. ST-RNA seq, spatial transcriptomics sequencing. GEO, Gene Expression Omnibus.
