# Supplementary material for: Single-cell multi-omics deciphers the myofibro-inflammatory program of cancer-associated fibroblasts in triple-negative breast cancer
Source: Cell Death Discov. 2026 Jul 11;12:404. doi: 10.1038/s41420-026-03255-z (PMC13642949; doi:10.1038/s41420-026-03255-z)
Supplement: Supplementary file 2 [file 41420_2026_3255_MOESM2_ESM.docx]

**Supplemental Figures**

**Supplemental Figure 1. The my_iCAFs promote TNBC progression.**

(a) Flowchart: Isolation and culture of my_iCAFs. (b) Western blot analysis for characterization of my_iCAFs. (c-d) EDU assay: Proliferation levels and quantification of breast cancer cells after co-culture with my_iCAFs or NFs. (e-f) Mouse subcutaneous tumor formation assay: In vivo tumor formation levels of breast cancer cells after co-culture with my_iCAFs or NFs, with quantitative comparison. (g) Genes associated with technical noise were filtered out prior to deconvolution using the Bayesian inference framework. (h) Comparison of gene expression patterns between bulk RNA-seq data and the scRNA-seq. (i) Prognostic significance of deconvoluted my_iCAF levels. Patients in TCGA-BRCA cohort were stratified by estimated my_iCAF abundance.

**Supplemental Figure 2. FOSB as a transcription factor activates HES1 mRNA transcription level.**

(a) JASPAR database analysis of potential motifs of FOSB. (b) qRT-PCR detection of the reversal effect of FOSB knockdown in my_iCAFs on CXCL8-induced upregulation of HES1 expression. (c) ChIP assay was designed based on the predicted FOSB binding domain on the HES1 mRNA promoter sequence (top), and qPCR was used to validate FOSB binding to the HES1 promoter region (bottom).
